# Surgical Management of Ovarian Endometrioma: Impact on Ovarian Reserve Parameters and Reproductive Outcomes

**DOI:** 10.3390/jcm12165324

**Published:** 2023-08-16

**Authors:** Angelos Daniilidis, Georgios Grigoriadis, Dimitrios Rafail Kalaitzopoulos, Stefano Angioni, Üzeyir Kalkan, Adrien Crestani, Benjamin Merlot, Horace Roman

**Affiliations:** 1 1st Department in Obstetrics and Gynaecology, Papageorgiou General Hospital, School of Medicine, Aristotle University of Thessaloniki, 54643 Thessaloniki, Greece; angedan@hotmail.com; 2 2nd Department in Obstetrics and Gynecology, Hippokratio General Hospital, School of Medicine, Aristotle University of Thessaloniki, 56429 Thessaloniki, Greece; drgeorgiosgrigoriadis@gmail.com; 3 Department of Obstetrics and Gynecology, Cantonal Hospital of Schaffhausen, 8208 Schaffhausen, Switzerland; dimkal1991@windowslive.com; 4 Department of Obstetrics and Gynecology, University of Cagliari, Monserrato, 09042 Cagliari, Italy; sangioni@yahoo.it; 5 Department of Obstetrics and Gynecology, Koç University, 34010 Istanbul, Turkey; uzekal@hotmail.com; 6 Institut Franco-Europeen Multidisciplinaire d’Endometriose (IFEMEndo), Endometriosis Centre, Clinique Tivoli-Ducos, 33000 Bordeaux, France; adriencrestani@hotmail.fr (A.C.); merlot.benjamin@gmail.com (B.M.); 7 Franco-European Multidisciplinary Endometriosis Institute (IFEMEndo), Middle East Clinic, Burjeel Medical City, Abu Dhabi 7400, United Arab Emirates; 8 Aarhus University, 8000 Aarhus, Denmark

**Keywords:** endometrioma, endometriosis, cystectomy, laser, sclerotherapy, fertility, pregnancy

## Abstract

Ovarian endometriomas have a negative impact on a patient’s reproductive potential and are likely to cause a reduction in ovarian reserve. The most commonly employed ovarian reserve parameters are anti-Müllerian hormone (AMH) and antral follicular count (AFC). Surgical management options of endometrioma include cystectomy, ablative methods, ethanol sclerotherapy and combined techniques. The optimal surgical approach remains a matter of debate. Our review aimed to summarize the literature on the impact of surgical management of endometrioma on AMH, AFC and fertility outcomes. Cystectomy may reduce recurrence rates and increase chances of spontaneous conception. However, a postoperative reduction in AMH is to be anticipated, despite there being evidence of recovery during follow-up. The reduction in ovarian reserve is likely multi-factorial. Cystectomy does not appear to significantly reduce, and may even increase, AFC. Ablative methods achieve an ovarian-tissue-sparing effect, and improved ovarian reserve, compared to cystectomy, has been demonstrated. A single study reported on AMH and AFC post sclerotherapy, and both were significantly reduced. AMH levels may be useful in predicting the chances of conception postoperatively. None of the aforementioned approaches has a clearly demonstrated superiority in terms of overall chances of conception. Surgical management of endometrioma may, overall, improve the probability of pregnancy. Evidence on its value before medically assisted reproduction (MAR) is conflicting; however, a combination of surgery followed by MAR may achieve the optimal fertility outcome. In view of the complexity of available evidence, individualization of care, combined with optimal surgical technique, is highly recommended.

## 1. Introduction

Endometriosis is a common gynecological disease, affecting an estimated 20 to 50% of the infertile population [1]. We recognize three phenotypes of endometriosis: superficial peritoneal lesions (SUP), deep endometriosis (DE), and ovarian endometrioma (OMA) [2]. OMA accounts for 17% to 44% of all cases [3], and is often diagnosed as part of the investigations for pelvic pain and/or infertility.

The presence of OMA may require surgical management, particularly in symptomatic women. The precise effect surgical management of OMA may have on ovarian reserve parameters and reproductive outcomes remains a matter of debate.

### 1.1. Ovarian Reserve

This is defined as the woman’s fertility potential at a given time and is determined by the primordial follicles that can develop into primary, antral and ovulatory follicles [4]. Factors such as aging, genetic defects, autoimmune disorders, chemotherapy and radiotherapy may negatively impact ovarian reserve [5]. Ovarian surgery, particularly for OMA, may lead to a postoperative reduction in the ovarian reserve [6,7].

Various measures have been used to assess the follicular pool. These include endocrine markers such as anti-Müllerian hormone (AMH), follicle stimulating hormone (FSH) and inhibin B, and ultrasound markers such as antral follicular count (AFC), ovarian volume and ovarian pulsatility and resistance indexes. In recent years, AMH and AFC have emerged as the preferred methods of assessing ovarian reserve [4].

AMH is produced by the granulosa cells of the primary, pre-antral and antral follicles and regulates the number of primordial follicles that enter the maturation process, thus preventing premature exhaustion of the ovarian reserve [8]. Its measurement offers objectivity, reproducibility and stability throughout the menstrual cycle. However, it is labor intensive, there is no standardization across assays, and it does not allow for the precise estimation of the effect of unilateral ovarian surgery. AFC, measured by transvaginal ultrasound during the follicular phase, is the total number of follicles measuring 2–10 mm. It is fairly easy to perform, provides immediate results and is independent of compensation by the unoperated healthy ovary. Its disadvantages include intra- and inter-cycle variation, differences in measurements between clinicians due to subjectivity and need for vaginal access.

Several studies have suggested that the presence of OMA per se is associated with lower AMH levels compared to women without OMA [9,10,11] or non-endometriotic ovarian cysts [9], as well as a more significant AMH decline over time compared to healthy, age-matched controls [12]. However, a cross-sectional study found only age and prior ovarian surgery (not the presence of OMA per se) to be linked to AMH levels < 1 ng/mL [6]. Nieweglowska et al. found bilateral, but not unilateral, OMAs to have a significant negative association with reduced preoperative AMH levels [13].

AFC has been found to be lower in ovaries with OMA [10,14], possibly due to inflammation [15], or the inability to perform a correct count due to anatomic distortion caused by the OMA [16]. However, a systematic review and meta-analysis found women with intact OMAs to have similar AFC to those without [17]. Lima et al. found ovaries with OMA to have lower AFC than unaffected ones; however, the number of oocytes retrieved was similar, suggesting a possible underestimation due to impaired visualization [18]. Inal et al. observed that women with OMA undergoing in vitro fertilization–intracytoplasmic sperm injection (IVF-ICSI) had, compared to controls, lower AFC and number of oocytes retrieved, despite similar AMH levels, concluding that AFC is an optimal marker of ovarian response in women undergoing IVF-ICSI [19].

### 1.2. Conservative Surgical Management Options

Conservative surgical management of OMAs generally involves the following options: cystectomy by stripping, ablative approaches (using laser, plasma energy or bipolar diathermy), sclerotherapy with ethanol and combined approaches. Advances in available technology and training have enabled the vast of majority of these procedures to be performed, nowadays, through minimal-access routes (i.e., laparoscopy or robot-assisted laparoscopy).

Cystectomy by stripping begins by draining the OMA and then gently pulling apart the cyst wall from the ovarian cortex, while applying careful traction and counter-traction. Hemostasis is then applied to the ovarian cyst bed, often by the use of bipolar diathermy, sutures and/or hemostatic agents.

Ablative techniques involve fenestration, drainage, wash-out and subsequent destruction of the OMA cyst wall. Endometriotic tissue is only present at a mean depth of 0.6 mm [20]; therefore, ablation of the entire depth of the cyst wall is not required, and may prove detrimental to the ovarian reserve. Compared to bipolar diathermy, laser and plasma energy achieve a more shallow, tissue-sparing effect, thus minimizing the inadvertent damage to the underlying ovarian parenchyma [21,22]. Plasma energy induces formation of a thin coagulum, sealing the tissue surface at a depth seldom exceeding 0.6 mm [23].

In large OMAs, the ‘three-stage procedure’ may be employed: Initial drainage of the cyst during laparoscopy is followed by 12 weeks of GnRH-agonist administration to reduce cyst size and then, laser vaporization of the cyst wall during a second laparoscopy [21,24].

Donnez et al. described a combined approach with cystectomy of 80–90% of the OMA and laser ablation of the remaining 10–20% close to the hilus, with the aim of minimizing inadvertent follicular loss [25]. Modifications to the combined approach have been described [26,27].

Sclerotherapy of OMA by laparoscopy involves puncture of the cyst, aspiration of its content and exposure of the cyst wall to 96% alcohol solution (ethanol) for 10 to 15 min [28].

Traditionally, cystectomy has been considered the gold- standard surgical approach, owing to the reduced risk of recurrence of OMA and endometriosis-associated pain [29], and increased chances of spontaneous conception [30]. However, it may lead to a reduction in postoperative ovarian volume [31], and may be associated with a risk of ovarian failure of 2.4% [32]. Therefore, the aforementioned surgical techniques have emerged as valid alternatives to cystectomy.

The aim of this narrative review is to present the evidence on how the different surgical approaches to OMA impact ovarian reserve parameters (namely, AMH and AFC), chances of spontaneous conception and medically assisted reproduction (MAR) success.

## 2. Materials and Methods

We performed a systematic electronic search on PubMed and MEDLINE databases, using the keywords “endometrioma”, “ovarian reserve”, “anti-Müllerian hormone”, “AMH”, “antral follicular count”, “AFC”, “surgery”, “fertility”, and “pregnancy”. We limited the search to English-language articles published between 2010 and 2023. Abstracts presented in scientific meetings were excluded.

Abstracts were reviewed by one of the authors (G.G.) and, if considered appropriate, the full texts of all potentially eligible studies were retrieved and reviewed in detail by the same author. A second author (A.D.) further reviewed all studies, and studies were included in this narrative review only if both authors agreed. There was no disagreement between the two reviewers in the selection of studies to be included. Had there been a disagreement, a third reviewer would have been consulted.

We decided to divide the “Results” section of our review into two main parts: The first part is focused on the impact of conservative surgical management options on AMH and AFC. The second part is focused on the reproductive outcomes following conservative surgical management options. We also included tables on each surgical management option, including all publications deemed relevant, with a focus on pregnancy rates (spontaneous and through MAR), recurrence rates, and AMH and AFC values.

## 3. Results

### 3.1. AMH

#### 3.1.1. Cystectomy and AMH

A systematic review of 11 studies suggested that OMA cystectomy was associated with a significant reduction in ovarian reserve, as demonstrated by a drop in AMH [33]. The deleterious effect of cystectomy on AMH was confirmed by a systematic review and meta-analysis of eight prospective cohort studies [7], which reported a 38% decline in AMH levels with a weighted mean difference of −1.13 ng/mL (95% confidence interval: −1.88 to −0.37). However, the level of heterogeneity of the included studies was high for both systematic reviews.

Celik et al. found, in their prospective study, that AMH levels were significantly reduced at 6 months post cystectomy compared to baseline (from 1.78 ± 1.71 to 0.72 ± 0.79 ng/mL; *p* < 0.001) [34]. Patients with cyst size ≥5 cm had a more profound decrease in AMH compared to those with OMA < 5 cm (65.7% vs. 41.3%). Alborzi et al. found OMA cystectomy to be associated with a significant decrease in AMH at 1 week (*p* < 0.001), 3 months (*p* < 0.001), and 9 months (*p* < 0.001) postoperatively compared to baseline. The decline was higher for those with bilateral OMAs and those who were older than 38 years [35]. In a prospective cohort study of 116 women, OMA cystectomy led to a significant drop in AMH at 1 month after surgery, with the levels remaining non-significantly lower at 6 months (baseline: 1.77 (1.18–2.37); 1 month: 1.12 (0.81–1.45); 6 months: 1.41 (0.97–1.85); *p* = 0.22] [11]. Lee et al. found cystectomy to cause a significant reduction in AMH levels for 3 months postoperatively, and the pattern of change was similar to that of oophorectomy (*p* = 0.002 for cystectomy and *p* < 0.001 for oophorectomy) [36]. Cystectomy led to a sustained reduction in AMH during the 9-month follow-up period (baseline: 3.0 ± 0.4; 3 months: 1.4 ± 0.2; 9 months: 1.3 ± 0.3, <0.0001) in Biacchiardi’s study [37]. In a prospective study of 104 patients, OMA cystectomy led to significant decrease in AMH for up to 12 months postoperatively (baseline: 3.77 ng/mL; 12 months: 1.72 ng/mL). In bilateral OMAs, the rate of decrease remained unchanged for 12 months, but in the unilateral group, it was lower at 12 months compared to at 6 months. The size of the OMA, bilaterality and baseline AMH seemed to be independent predictors of AMH level at 12 months [38].

Various studies have suggested a recovery in AMH during follow-up: Sugita et al. performed a prospective study including 39 patients and observed that 20 of those had higher AMH levels at 1 year after cystectomy than at 1 month (increase group), and 19 had lower AMH at 1 year (decrease group) [39]. Interestingly, in the increase group, the follicular loss during surgery was higher (*p* = 0.035), with the authors suggesting that mechanisms other than follicular loss may be involved in cases of sustained ovarian reserve reduction. A prospective study with 171 patients showed that, 12 months after surgery, AMH levels were no different from the preoperative assessment in OMAs ≤ 7 cm, unilateral cysts, and stage 3 endometriosis [40]. Kostrzewa et al. observed a significant decrease in AMH levels 3 months after cystectomy (4.89 ± 3.66 vs. 3.45 ± 3.37 ng/mL; *p* < 0.001), but no further fall in the 1-year assessment (3 months = 3.45 ± 3.37 vs. 1-year = 3.43 ± 3.62 ng/mL; *p* > 0.05) [41]. In a prospective, follow-up study, there was no significant difference in AMH levels (compared to baseline) at 6 and 12 months after OMA cystectomy, despite a significant drop in the first month postoperatively. It is worth noting that hemostasis was achieved by sutures only, with no use of bipolar diathermy [42]. Kovačević et al. observed that AMH levels at 6 and 12 months after OMA cystectomy were significantly lower compared to baseline, both for unilateral and bilateral OMAs. When examining the issue of ovarian reserve recovery, they noted a borderline significant recovery in the unilateral OMA group (*p* = 0.056), whereas some recovery, but not significant, was noted in the bilateral group (*p* = 0.698). It is worth noting that the majority of patients received medical therapy immediately postoperatively, but no therapy between 6 and 12 months [43]. In another prospective study, AMH levels decreased at 1, 3 and 6 months postoperatively; however, there was no difference between baseline AMH levels and those at 12 months after surgery (baseline: 3.98 ± 3.27 ng/mL, 12 months: 4.01 ± 3.39 ng/mL) (*p* > 0.05). The rate of decline was higher for bilateral OMAs compared to unilateral, 12 months after cystectomy (*p* = 0.035) [44].

In a prospective, randomized clinical trial, both OMA cystectomy and deroofing decreased AMH levels at 12 months postoperatively; however, the reduction was more significant in the cystectomy group (−2.59 ± 1.05 change in the cystectomy group, −2.13 ± 0.95 in the deroofing group; *p* = 0.012) [45].

In their systematic review and meta-analysis of prospective studies comparing the effect of unilateral vs. bilateral OMA cystectomy on ovarian reserve, Younis et al. found postoperative AMH drop to be more significant in the bilateral cystectomy group and maintained one year after surgery. In both groups, postoperative AMH levels were lower compared to baseline, despite a non-significant difference in AMH between unilateral and bilateral OMAs before surgery [46]. According to Raffi et al., excision of bilateral OMAs led to higher AMH decline, compared to unilateral cystectomy (44% vs. 30%); however, the difference was not significant, possibly due to the small number of patients in the included studies [7].

In a retrospective study, OMA cystectomy did not lead to a significant difference in the values of FSH and AMH at 3 months compared to baseline (FSH(U/mL): baseline: 7.3 ± 1.3; 3 months: 9.0 ± 1.2; *p* = 0.202. AMH (ng/mL): baseline: 3.3 ± 0.5; 3 months: 2.1 ± 0.3; *p* = 0.321) [47]. Similarly, Ercan et al. did not observe a significant decrease in AMH at 3 months after unilateral OMA cystectomy (baseline: 2.03 ± 0.41 ng/mL vs. 1.95 ± 0.62 ng/mL; *p* > 0.05) [48].

Kitajima et al. compared postoperative AMH levels following conventional, one-step cystectomy vs. their “three-step” approach (initial fenestration and drainage of OMA, followed by 3 months of oral dienogest, and then second-look laparoscopy with cystectomy) in a prospective study of 12 women [49]. At 9–12 months postoperatively, AMH levels in the “three-step” approach did not differ significantly from baseline (*p* = 0.16), in contrast to one-step cystectomy, where they remained lower (*p* = 0.01). They also observed proinflammatory cytokines and chemokines to be downregulated in the peritoneal fluid of women following the “three-step” approach compared to in those using the one-step approach, suggesting that dienogest may exert its effect partly through alleviating inflammation.

A recent randomized controlled trial linked peri-operative use of dienogest for a total of 4 months with better ovarian reserve preservation compared to GNRH analogue use following OMA cystectomy: in the dienogest group, after 1 year of follow-up, >60% of patients retained over 70% of baseline AMH levels, compared to no patients in the GNRH analogue group (*p* < 0.01). Moreover, women in the dienogest group had lower IL-6 levels at the end of treatment, a finding that supports dienogest’s anti-inflammatory action [50].

#### 3.1.2. Ablative Methods and AMH

In a prospective study by Roman et al., plasma energy ablation of OMA led to a significant decrease in AMH levels at 3 months after surgery, followed by an increase at >6 months (3.9 ± 2.6 ng/mL before the surgery, 2.3 ± 1.1 ng/mL at 3 months, and 3.1 ± 2.2 ng/mL at the end of the follow-up (*p* = 0.001)). Τhe levels remained lower compared to baseline, albeit not significantly. The authors postulated that, taking into account that plasma energy ablation spares ovarian parenchyma, other factors such as inflammation may have led to the unexpected decline in AMH [51].

#### 3.1.3. Laparoscopic Sclerotherapy and AMH

In a retrospective study of 69 women, AMH significantly decreased (from 3.4 ng/mL (SD 2.3) before surgery to 2 ng/mL (SD 1.7) after surgery (*p* < 0.001)) following laparoscopic sclerotherapy of OMA with 95% ethanol during a follow-up of 11.5 ± 4.6 months. Exposure time > 10 min was not associated with reduced risk of recurrence [52].

#### 3.1.4. Cystectomy vs. Ablative Methods and AMH

In Saito’s study, the rate of AMH decline was significantly higher at 1 month, 6 months and 12 months after bilateral OMA cystectomy than for cyst vaporization with bipolar diathermy (1 month, *p* = 0.04; 6 months, *p* = 0.02; 1 year, *p* = 0.02). No statistically significant differences were noted between the two surgical approaches in the case of unilateral OMA. The authors observed that both approaches had the potential to lower AMH levels, particularly in patients older than 38 years and those with severe endometriosis [53].

In a prospective randomized study, traditional OMA cystectomy was compared with a modified technique of stripping combined with bipolar coagulation of the cyst wall and cutting close to the ovarian hilum. At 1 month postoperatively, AMH was significantly reduced in both groups, with the drop in AMH being higher in the traditional cystectomy group (9.1% vs. 4.5%) [54].

Chen et al., in their retrospective study, found that OMA cystectomy led to a greater AMH decline compared to the drainage and bipolar coagulation of the OMA cyst wall (cystectomy: 0.85 ± 0.64 vs. drainage/bipolar: 0.52 ± 0.58; *p* = 0.04). Recurrence rates were lower, but not significantly, following cystectomy [55].

In a small randomized clinical trial of 60 patients with 3-month follow-up, cystectomy led to a greater decline in AMH (from 2.6 ± 1.4 ng/mL at baseline to 1.8 ± 0.8 ng/mL at 3-month follow-up; 95% CI: −1.3 to −0.2; *p* = 0.012), while no reduction was observed in the one-step CO_2_ laser group (from 2.3 ± 1.1 ng/mL at baseline to 1.9 ± 0.9 ng/mL at 3-month follow-up; 95% CI: −1 to −0.2; *p* = 0.09) [56]. In a case–control study with 2-week follow-up, 40 patients underwent argon-beam laser coagulation and 40 patients underwent cystectomy for OMA. There was no statistically significant difference in AMH levels between the two groups, either before (8.8 ± 4.5 vs. 8.9 ± 4.5; *p* = 0.9) or after (2.65 ± 1.38 vs. 3.0 ± 2.1; *p* = 0.36) intervention. However, in both groups, postoperative AMH levels were significantly lower than baseline [57]. Tsolakidis et al., in their prospective randomized study, found that cystectomy for OMA led to a significant decrease in AMH (from 3.9 ± 0.4 to 2.9 ± 0.2; *p* = 0.026) compared to the “three-step” procedure (drainage of OMA, followed by 12 weeks of treatment with GnRH analogues and CO_2_ laser vaporization of the cyst wall) (from 4.5 ± 0.4 to 3.99 ± 0.6; *p* = NS). However, the decline in AMH was not paired with a reasonable decrease in inhibin B or E2 levels or a rise in FSH, as anticipated by the greater damage to ovarian reserve in the cystectomy group [58].

Giampaolino et al. found that both cystectomy and ablation with bipolar diathermy caused a significant decrease in baseline AMH, regardless of OMA size. For OMAs < 5 cm, they found no significant difference in AMH levels between the two surgical techniques (17.6 ± 4.7% vs. 18.2 ± 10.6%); however, for OMAs ≥ 5 cm, cystectomy showed a significantly greater decrease percentage compared with the ablation group (−24.1 ± 9.3% vs. −14.8 ± 6.7%; *p* = 0.011) [59].

### 3.2. AFC

#### 3.2.1. Cystectomy and AFC

In a systematic review and meta-analysis of 13 studies, despite a high level of heterogeneity, surgery was found not to lead to a significant difference in AFC levels (mean difference 0.10, 95% CI −1.45 to 1.65; *p* = 0.90). The AFC values of affected ovaries were lower compared to contralateral healthy ovaries, both before and after surgery; however, the difference was only significant postoperatively [14]. In Younis’s systematic review and meta-analysis [46], cystectomy for unilateral or bilateral OMA did not lead to a significant difference in AFC levels in either group compared to baseline. Only two studies that reported on AFC were included in the meta-analysis.

In a prospective study by Georgievska et al., despite a reduction in ovarian volume 3 months after OMA cystectomy, the AFC of the operated side was found to be significantly increased (baseline: 3 ± 1.34, 3 months: 5.48± 1.96; *p* < 0.001) and the FSH decreased, albeit not significantly [60]. OMA cystectomy led to a rise in AFC at both 6 weeks and 6 months after surgery (baseline: 4.9 ± 2.2; 6 weeks: 5.1 ± 2.4; 6 months: 6.4 ± 2.2; *p* = 0.008), according to Celik et al. [34]. Alborzi et al. reported that OMA cystectomy led to a significant rise in AFC at 3 months after surgery compared to baseline (from 7.81 ± 3.22 to 10.75 ± 3.68; *p* < 0.001) [35]. Bhat’s prospective study reported an increase in the AFC of the operated ovary at 1 month post OMA cystectomy (from 3.3 ± 1.9 to 4.1 ± 1.5; *p* = 0.001), with no significant difference in ovarian volume. Follicular loss was seen in 27.2% of specimens, and was significantly higher in OMAs < 5 cm compared to those >5 cm. Follicular loss was also higher in patients with moderate and severe disease, compared to those with minimal or mild disease; however, the difference was not statistically significant [61].

In Urman’s prospective study, following OMA cystectomy, the AFC decreased by 11% at 1 month (*p* = 0.01) and 15% at 6 months compared to baseline levels. Primordial follicles were found in 61.5% of specimens [62]. Sweed et al., in their prospective randomized study, found OMA cystectomy and deroofing to reduce AFC for up to 12 months postoperatively, with deroofing causing a smaller reduction (−5.7 ± 1.9 change in the cystectomy group, −1.6 ± 0.5 in the deroofing group; *p* < 0.001) [45]. In a study by Ercan et al., the mean AFC values of operated-side ovaries were significantly lower on the second postoperative day (3.1 ± 2.4 vs. 5.2 ±3.7; *p* < 0.05) and in the third month (3.7 ± 2.1 vs. 6.4 ± 2.7; *p* < 0.05), albeit with no significant impact on ovarian volume and Doppler indices [48].

Kostrzewa et al. reported no difference in AFC during 12 months of follow-up after OMA cystectomy [41]. AFC did not significantly change during the 9-month follow-up (baseline: 3.3 ± 3.2; 3 months: 6.0 ± 4.5; 9 months: 5.1 ± 3.6, *p*-value: NS) in Biacchiardi’s prospective study [37]. In another prospective cohort study, AFC remained unchanged (9.7 ± 4.8 vs. 10.4 ± 4.2; *p* = 0.63) at 6 months after surgery [10]. Ding et al. found no significant difference in AFC levels at 6 and 12 months after OMA cystectomy compared to baseline, despite there being a significant decline at 1 month post procedure. Only sutures, and no bipolar diathermy, were used for hemostasis [42].

#### 3.2.2. Ablative Methods and AFC

In a prospective clinical trial of 15 patients, CO_2_ laser led to a significant increase in the AFC of the operated ovary at 1 and 3 months after surgery (*p* = 0.0021 and *p* = 0.005, respectively), which was particularly significant for women younger than 35 years [63].

In a retrospective study, plasma energy ablation of OMA led to a decrease in AFC and ovarian volume by an average of 18% and 12%, respectively, compared to the contralateral healthy ovary [64].

#### 3.2.3. Laparoscopic Sclerotherapy and AFC

Crestani et al. found laparoscopic sclerotherapy of OMA with 95% ethanol to lead to a non-significant increase in AFC compared to baseline (from 8.2 (SD 7) to 12 (SD 9.6); *p* = 0.9) [52].

#### 3.2.4. Combined Technique and AFC

The combined technique, including cystectomy of the largest part of the OMA and CO_2_ laser ablation of the remaining 10–20% close to the hilus, did not lead to a reduction in AFC or ovarian volume after 6 months of follow-up compared to women with non- endometriotic ovaries of the same age (AFC: combined technique: 6.1 ± 3.2; no endometriosis: 6.2 ± 4.8. Ovarian volume: combined technique: 7.64 ± 2.95; no endometriosis: 7.99 ± 5.33). Ovarian follicles were present in only one case (2%) [25].

#### 3.2.5. Cystectomy vs. Ablative Methods and AFC

In a prospective clinical trial with 60-month follow-up, in which cystectomy was compared with laser ablation, pregnancy rates, AFC and FSH levels were comparable at the end of follow-up. Recurrence rates were statistically higher in the laser group at 12 months and later, although they were not significantly different at the end of follow-up [65].

Candiani et al. found one-step CO_2_ laser to lead to a higher postoperative increase in AFC compared to cystectomy (CO_2_ laser: from 4.1 ± 2.2 (mean ± SD) at baseline to 6.3 ± 3.5 at 3-month follow-up; 95% CI: 0.9–4; cystectomy: from 3.6 ± 1.9 at baseline to 8.6 ± 4.2 at 3-month follow-up; 95% CI: 2.8–7.1; *p* = 0.016). At a mean follow-up of 5.3 months, pregnancy rates of 15.4% in the CO_2_ laser group and 25% in the cystectomy group were reported [56]. According to Gheit et al., both OMA cystectomy and laser ablation led the operated ovary having higher AFC compared to baseline, but the increase was significantly higher in the laser group [57].

In Pados’s randomized study, in which two approaches were compared, the “three-step” procedure was associated with a significant improvement in folliculogenesis compared to cystectomy, as demonstrated by AFC of the operated ovary after 6 months of follow-up (“three-step” procedure: from 1.27 to 4.36; cystectomy: from 2 to 2.38; *p* = 0.002). Interestingly, the two groups did not differ significantly with respect to postoperative ovarian volumes and ovarian vascularization [21].

In a prospective study of women with bilateral OMAs > 3 cm, of which one was randomly allocated to undergo cystectomy and the other CO_2_ laser vaporization, AFC and ovarian volumes 6 months after surgery were significantly higher in the laser group compared to cystectomy, despite there being no significant differences in either parameter at baseline. Three pregnancies occurred out of nine patients wishing to conceive postoperatively and, in all cases, the corpus luteum was on the ovary that had laser vaporization. No recurrences were reported [66].

In a retrospective study of 30 women managed for unilateral OMA > 30 mm, OMA cystectomy led to a significantly lower postoperative AFC compared to plasma energy vaporization (plasma: 5.5 ± 3.9; cystectomy: 2.9 ± 2.4; *p* = 0.03), which remained significant after adjustment for age, previous pregnancy and cyst diameter [22]. Georgievska et al. found that both laparoscopic cystectomy as well as puncture and endocoagulation led to a significant increase in AFC at 1 and 3 months after surgery compared to baseline. However, the AFC increase at 3 months was significantly higher in the puncture and endocoagulation group [60].

Both cystectomy and bipolar coagulation led to AFC reduction after 6 months of follow-up, with cystectomy causing a greater decline (*p* = 0.001), according to a prospective randomized study [67].

#### 3.2.6. Cystectomy vs. Combined Technique and AFC

In a multicenter, randomized clinical trial of 51 patients with bilateral OMAs, one side was allocated to stripping cystectomy and the other to a combination of stripping (80–90% of the cyst) and ablation by bipolar coagulation (10–20% of the cyst, close to the hilum). Postoperative AFC at 6 months did not differ between the two approaches (stripping: 4.8 ± 2.9; combined technique: 4.4 ± 2.3; *p* = 0.57) [68].

### 3.3. Fertility Outcomes

#### 3.3.1. Fertility Outcomes and OMA Presence

OMAs per se appear to be linked to reduced reproductive potential [69]. It has been demonstrated that the presence of OMA may have a negative effect on spontaneous ovulation [70], as well as follicular number and activity of the adjacent ovarian cortex [71].

It has been postulated that increased endometrial expression of the nuclear factor-kappa B (NF-kB) may be associated with endometriosis-related infertility and surgical excision of OMA may lead to a reduction in the expression of NF-kB1 and NF-kB p65 in the eutopic endometrium [72]. The concept of improved endometrial receptivity following laparoscopic OMA excision was further supported by a recent case–control study [73].

The concurrent presence of OMA reduces the chances of spontaneous conception in women with rectovaginal endometriosis, regardless of expectant or surgical management, according to a retrospective study by Maggiore et al. [74].

As regards the impact of ovarian OMA on ovarian responsiveness to stimulation and the outcome of assisted reproduction, a meta-analysis by Gupta et al. found that presence of OMA was associated with decreased ovarian response to ovarian stimulation, possibly due to reduced number of follicles. However, the odds for clinical pregnancy and the overall pregnancy rate did not differ significantly with controls [75].

Yang et al., in their systematic review and meta-analysis of nine studies, found that women with OMA had significantly lower number of oocytes retrieved (mean difference (MD) −1.50; 95% CI, −2.84 to −0.15; *p* = 0.03), metaphase II (MII) oocytes retrieved (MD −3.61; 95% CI −4.44 to −2.78; *p* < 0.00001) and total embryos formed (MD −0.66; 95% CI −1.13 to −0.18; *p* = 0.007) compared to controls. However, intra-patient comparisons in patients with unilateral OMA found no significant difference in total number of oocytes, MII oocytes retrieved, or total embryos formed between the affected and healthy gonad. Furthermore, total gonadotrophin use, stimulation duration, embryo quality, implantation rate, clinical pregnancy rate and livebirth rate were similar between women with OMA and controls [76].

Other studies have also suggested that the presence of OMA per se has a negative impact on AFC and response to ovarian stimulation [77,78].

However, in a study by Esinler et al., the number of oocytes retrieved from ovaries with OMA ≤ 3 cm was comparable to that of contralateral, healthy ovaries (5.9 ± 4.3 vs. 5.4 ± 3.8), suggesting that smaller OMAs do not appear to impact negatively upon the ovarian reserve [79]. In a prospective study by Filippi et al., the presence of unoperated, unilateral OMA did not affect the ovarian responsiveness or retrieved oocyte quality in women undergoing IVF compared to the healthy contralateral gonad. The fertilization rate was the same (64%) between the affected and healthy gonads [80].

In patients with diminished ovarian reserve (D.O.R.) (AMH < 1.1 ng/mL), the presence of OMA or not does not impact on IVF outcomes and time to achieve livebirth, according to a retrospective case–control study [81].

In a retrospective study by Dong et al., patients with unoperated OMAs undergoing IVF/ICSI had lower AFC and required higher doses of gonadotrophins; however, the CPR and ongoing pregnancy rate/LBR did not differ from those in women who had undergone laparoscopic OMA cystectomy and had no visible recurrence [82].

Wu et al. found OMA to be associated not only with reduced oocyte quantity, but also with poorer quality, in women undergoing IVF/ICSI. However, they observed no significant impact on overall pregnancy outcomes [83].

According to Liang et al., ovarian OMAs are not associated with cytokine profiles in the follicular fluid of infertile women and are, therefore, unlikely to affect oocyte and embryo quality through inflammation [84].

A large retrospective cohort study of 2245 infertile women found that women with ASRM stage 3 or 4 endometriosis and OMA, necessitated more FSH and had a significantly lower pregnancy and live birth/ongoing pregnancy rate, compared to those with ASRM stage 3 or 4 endometriosis but without OMA [85].

In a 5-year retrospective study of 619 patients, those with pelvic endometriosis and OMA (N = 398) undergoing IVF-ET had fewer oocytes retrieved and 2-pronuclei embryos in all age groups (*p* < 0.01) compared to patients with pelvic endometriosis without OMA. As the number of oocytes and 2-pronuclei embryos were important predictors of IVF-ET success, the authors concluded that the presence of ovarian OMA exerts a negative impact on IVF-ET efficacy [86].

#### 3.3.2. Fertility Outcomes and OMA Surgery

In a systematic review and meta-analysis of prospective studies of women with infertility and OMA, pregnancy rates were compared between four approaches: surgery + ART, surgery + spontaneous pregnancy, aspiration ± sclerotherapy + ART, and ART alone. The success of surgery was higher (43.8%, CI: 22.5–66.4), and IVF alone led to a lower clinical pregnancy rate (32%, CI: 15.0–52.0); however, the differences were not significant [87].

Raffi et al. described a total pregnancy rate after OMA surgery of 71%, which is significantly lower than the 98% natural pregnancy rate in the control group (*p* = 0.0001). OMA surgery, however, significantly improved the chances of success of fertility treatments from 7% to 63% (*p* = 0.001) [69].

Surgical excision of OMA does not exert a qualitative impact on oocytes retrieved, according to a retrospective study by Harada et al. [88]; compared to oocytes from healthy ovaries, those from ovaries with history of cystectomy did not differ in terms of fertilization rate (63.6 % vs. 69.5 %; *p* = 0.43) or rate of top-quality embryos (40.0 % vs. 49.0 %; *p* = 0.34). Clinical and ongoing pregnancy rates per embryos were also similar.

#### Cystectomy and Pregnancy Rates

Taniguchi et al. reported a 50% pregnancy rate (of which 50% were spontaneous) following OMA cystectomy. Notably, the postoperative decline rate of AMH levels at 1 year in the patients who achieved spontaneous pregnancy was significantly lower than that in the patients with infertility treatment [89].

The pregnancy rate post OMA cystectomy was 77.4% (24 out of 31 patients) in Kovačević’ s study, of which 75% (18 out of 24) were spontaneous conceptions [43].

#### Ablative Techniques and Pregnancy Rates

In a retrospective study with minimum 1 year follow-up, plasma ablation of OMA led to postoperative pregnancy in 67% of women who wished to conceive, with spontaneous conception in 59% of those cases. The recurrence rate was 10.9% [90].

A prospective study identified a 73% total pregnancy rate following plasma ablation of OMA; 37% of conceptions were spontaneous. One case of OMA recurrence was observed during follow-up (5%) [51].

A total pregnancy rate of 61.4% (of which 64.7% was spontaneous) was reported in a prospective study of women with OMAs managed by plasma energy ablation, with or without colorectal endometriosis. Of interest, management of colorectal endometriosis at the time of OMA management did not appear to impact the chances of pregnancy or recurrence rate, which was reported at 14.5% for the whole study population [91].

Lockyer et al., in their retrospective study of women with unilateral or bilateral OMAs ≥ 25 mm in diameter, with associated pelvic pain and/or infertility managed by plasma energy ablation reported a pregnancy rate of 46.2% (6 of the 13 women wishing to conceive fell pregnant), all of them by MAR techniques. A recurrence rate of 9.5% was observed. The study showed a statistically significant decrease in the proportion of patients reporting dysmenorrhea, dyspareunia, and chronic pelvic pain postoperatively [92].

#### Laparoscopic Sclerotherapy and Pregnancy Rates

Crestani et al., in their retrospective study of 69 women managed by laparoscopic sclerotherapy of OMA with 95% ethanol, reported a 40.1% postoperative pregnancy rate, with 61% through ART. Recurrences were recorded in 11.8% of cases during a follow-up of 11.5 ± 4.6 months. Exposure time >10 min was not associated with reduced risk of recurrence [52].

Another retrospective study of laparoscopic ethanol sclerotherapy of OMA reported a 57% total pregnancy rate postoperatively, with 87% of those being spontaneous conceptions. The recurrence rate was 3% for those that received postoperative hormonal therapy for more than 6 months and 21% for those that received only for 3 months [93].

#### Combined Techniques and Pregnancy Rates

Donnez et al. reported a pregnancy rate of 41% during a mean follow-up of 8.3 months after their combined technique for OMA management [25].

Supermaniam et al. described their combined technique, which included injection of diluted vasopressin between the cyst wall and ovarian cortex, stripping of the cyst wall until close to the ovarian hilus, minimal bipolar ablation to the remaining cyst wall and ovarian reconstruction with suturing. They employed this technique in 143 patients with co-existent stage 3 or IV endometriosis. The primary outcome was clinical pregnancy rate. Out of 76 patients with preoperative infertility and pregnancy intention, 38 (50%) were successful in achieving a pregnancy. A total of 32 patients achieved spontaneous conception within a mean duration of 6.9 months of trying to conceive, 5 patients via IVF/ICSI and 1 patient following ovulation induction. Patients who underwent bilateral cystectomy had lower pregnancy rate compared to unilateral cystectomy. The spontaneous pregnancy rate was also lower in patients with severe endometriosis as compared to moderate endometriosis, 34.6% vs. 62.5% respectively [27].

#### Comparing Surgical Approaches and Pregnancy Rates

In a meta-analysis of seven randomized controlled trials, cystectomy was associated with higher chances of postoperative conception compared to fenestration/coagulation but not to laser vaporization. Cystectomy also led to lower OMA recurrence rates compared to the other two approaches [94].

A multi-centric, prospective, case–control study compared postoperative pregnancy probability between women with OMA managed by cystectomy vs. plasma energy [95]. The probability of pregnancy at 24 and 36 months after plasma energy vaporization and cystectomy was respectively 61.3% (95% CI 48.2–74.4%) vs. 69.3% (95% CI 54.5–83%) and 84.4% (95% CI 72–93.4%) vs. 78.3% (95% CI 63.8–90%). Both approaches led to comparable pregnancy rates, and analysis showed the type of surgical procedure not to have a statistically significant impact on probability of pregnancy.

Puscasiu et al. recently reported the results of their retrospective, three-arm study, comparing postoperative pregnancy rates between OMA cystectomy, ablation by plasma energy and simple OMA drainage [96]. The overall postoperative pregnancy rate was 60.3%, with the probability of pregnancy at 12 months being 27% (cystectomy), 32% (plasma) and 16% (drainage). There was a statistically significant difference in pregnancy rates between the groups (*p* = 0.015). The proportion of spontaneous conceptions was 58% (cystectomy), 43% (plasma) and 27% (simple drainage).

Traditional OMA cystectomy led to a 10% postoperative pregnancy rate, which is similar to the pregnancy rate following a combination of cystectomy and bipolar coagulation of the cyst wall and cutting close to the ovarian hilum (9%) [54].

Chen et al., in their retrospective study, found cystectomy and drainage followed by cyst wall ablation by bipolar energy linked with similar pregnancy rates (71.05% following cystectomy and 73.08% following drainage and bipolar ablation, with a mean follow-up of 30.40 months and 32.35 months (*p* > 0.99), respectively) [55].

#### 3.3.3. OMA Surgery pre MAR

In a systematic review and meta-analysis, Hamdan et al. found that, compared with women with no surgical treatment, women who had their OMA surgically treated before IVF/ICSI had a similar LBR (OR: 0.90;95% CI [0.63, 1.28]), a similar CPR (OR 0.97; 95% CI [0.78, 1.20]) and a similar mean number of oocytes retrieved (SMD—0.17; 95% CI [−0.38, 0.05]) [17]. Two more meta-analyses reported similar outcomes in terms of clinical and livebirth rates, between surgically and conservatively managed OMAs pre IVF [97,98].

Tao et al. conducted a meta-analysis of 21 published studies (2649 Artificial Reproductive Technology cycles) assessing the role of OMA cystectomy pre IVF [99]. Women with a history of cystectomy required higher doses of gonadotrophins, had lower numbers of oocytes retrieved, and similar stimulation durations, total numbers of embryos formed, and pregnancy and livebirth rates compared to women that were managed by IVF only.

Compared to use of Gnrh-agonist only, a prospective clinical trial by Hosseinimousa et al. found that OMA cystectomy followed by Gnrh-agonist use led to a higher pregnancy rate (chemical and clinical) and live birth rate, albeit not statistically significantly. There was no difference in duration of stimulation, number of oocytes retrieved or number of embryos [100].

Age < 35, AFC > 7 and having two embryos transferred may be associated with better outcome in fresh embryo transfer following IVF/ICSI in women who had previously undergone OMA cystectomy [101].

In a retrospective study by Tang et al., OMA cystectomy pre-IVF led to significantly lower AFC and number of dominant follicles and oocytes retrieved. The effect was more significant for OMAs > 4 cm than those < 4 cm, suggesting that the size of OMA may play a role in the degree of ovarian damage caused by cystectomy [102].

A retrospective case–control study by Motte et al. found that, compared with controls, women with OMAs that underwent plasma energy ablation prior to IVF/ICSI had lower number of oocytes but better implantation rates, pregnancy and delivery rates per cycle, and cumulative birth rates per transfer [103].

There is concern regarding the risk of surgery-induced ovarian damage, leading to gonadal unresponsiveness during hyperstimulation. Benaglia et al. found that OMA cystectomy led to subsequent absence of follicular growth during hyperstimulation in 13% of cases (95% CI: 7–21%) [104].

Interestingly, a retrospective study of 45 women with previously operated OMA undergoing IVF found that ovaries with recurrent OMA had higher responsiveness compared to those without OMA recurrence (the mean ± SD number of follicles in gonads with and without recurrences was 2.5 ± 2.3 and 1.1 ± 1.5, respectively (*p* < 0.05)), presumably reflecting a more intact ovarian reserve [105].

D.O.R. following OMA cystectomy was linked to lower implantation rate and clinical and livebirth rate in IVF cycles compared to idiopathic D.O.R., according to Roustan et al. [106].

Those results contradict those reported in a retrospective study by Hong et al., according to which cases of D.O.R. following OMA cystectomy and those with idiopathic D.O.R. did not differ significantly in terms of clinical pregnancy and livebirth rate [107].

Cystectomy by “experienced” surgeons (attending physicians) led to a significantly higher postoperative AFC (9.6 ± 6.6 vs. 7.5 ± 3.8; *p* = 0.011) and livebirth rate per cycle (32.9% vs. 9.3%; *p* < 0.001) than that by “inexperienced surgeons” (chief residents and fellows) [108]. However, there was no significant difference between the two groups regarding mean number of oocytes, fertilization rate, mean number of embryos transferred, rate of good-quality embryos transferred, implantation rate, or clinical pregnancy.

In a retrospective study by Yu et al., the laterality of OMA (left or right side) did not impact ovarian reserve or IVF/ICSI outcome following laparoscopic cystectomy for unilateral OMA. However, the implantation rate (but not clinical or livebirth rate) was lower in women following left ovarian OMA cystectomy (10.1% vs. 20.2%; *p* = 0.015) [109].

Bilaterality of ovarian OMA does not appear to affect the outcome of IVF/ICSI post-cystectomy, according to a retrospective study by Yu et al. [110]. Implantation rates and clinical pregnancy, livebirth and miscarriage rates were similar between unilateral and bilateral OMAs, despite a significantly lower number of dominant follicles on the day of human chorionic gonadotropin (hCG) administration (5.2 ± 3.1 vs. 4.2 ± 2.7; *p* = 0.048) and a lower number of oocytes retrieved (10.0 ± 6.9 vs. 7.6 ± 6.6; *p* = 0.047) in the bilateral OMA group.

### 3.4. Tables of Surgical Approaches and Outcomes

The following tables summarize the published studies included in our review, based on the surgical approach utilized. Each table presents the evidence on one surgical approach, focusing on postoperative fertility outcomes, as well as AMH and AFC values. The fertility outcomes and AMH and AFC values given are at the end of follow-up, unless stated otherwise [Table 1, Table 2, Table 3, Table 4 and Table 5].

## 4. Discussion

Surgical management of OMA represents a major concern in the daily activity of surgeons involved in endometriosis management. Our study attempted to update and classify the abundant information that has been published in this field, with a spotlight on ovarian reserve conservation and the likelihood of pregnancy. Extensive information should be provided to patients preoperatively, in order to avoid further conflicts, particularly when it is planned for patients to undergo postoperative MAR and experience a decrease in AMH and AFC levels. For these reasons, we believe that our review could be of major interest to those colleagues interested in endometriosis surgery.

The presence of OMA may negatively impact ovarian reserve [9,10,11,14]. Its surgical management, on the other hand, may cause a further decline. Concerns, therefore, arise regarding the potential impact of various surgical management options on the fertility potential of affected women and the associated reproductive outcomes.

A postoperative reduction in AMH levels may be anticipated following any of the aforementioned surgical approaches; however, cystectomy is likely to be linked with the highest decline [51,52,53,54,55,56,58]. However, few studies have reported on long-term, postoperative follow-up and, based on those available, a gradual recovery with time in AMH levels may be anticipated [39,40,41,42,44]. The decline in ovarian reserve is probably multi-factorial and may be linked with inadvertent removal of healthy follicles at the time of cystectomy [126,127] and ovarian tissue damage caused by the use of bipolar diathermy. Various systematic reviews and meta-analyses have found bipolar diathermy to be more deleterious to ovarian reserve compared to non-thermal methods of hemostasis [128,129,130]. Other factors that may impact ovarian parenchymal loss and post-surgical ovarian reserve decline include OMA size [34,38,102,131,132], bilaterality [7,35,38,44,46], preoperative AMH levels [10,34,38,43], disease severity according to rASRM classification [132,133], and age [134,135]. Interestingly, the number of follicles inadvertently removed during cystectomy does not always correlate with the AMH decline after the procedure [115,133].

Can AMH levels be used to predict chances of spontaneous conception? According to Dong et al., the best cut-off point of preoperative AMH for postoperative spontaneous pregnancy is >3.68 ng/mL (Hazard ratio (HR): 2.383; 95% CI, 1.093–5.197) [136]. A very similar value was proposed by Zhou et al. (3.545 ng/mL; sensitivity 80.39%; specificity 69.23%) [137]. A retrospective study found no difference in the pregnancy rates between women with normal (≥2 ng/mL) and low (<2 ng/mL) AMH levels undergoing OMA ablation with plasma energy and excision of stage 3 and 4 endometriosis. Pregnancy rates were, respectively, 74.6% and 73.9%, while spontaneous conception represented 54% and 58.8% of the women in these cases [138]. In a study by Iwase et al., AMH levels 1 year after laparoscopic cystectomy for OMA were higher in women that got pregnant using infertility treatments than in non-pregnant women (3.44 ± 1.78 vs. 2.17 ± 2.24 ng/mL; *p* = 0.049). The authors concluded that AMH levels 1 year post cystectomy may be used to predict the success chances of postoperative fertility treatments, but not the chances of disease recurrence [139].

However, in a series of patients with severe endometriosis and preoperative AMH < 1 ng/mL, whose OMAs were treated by vaporization using plasma energy, postoperative pregnancy rate was as high as 68.2%, with 66.7% being spontaneous conception [138]. This study suggested that, in women with baseline low AMH and in which the results of the ART are expected to be sub-optimal, surgery could be a useful tool for improving natural conception, particularly when fallopian tubes and sperm parameters are normal.

Two systematic reviews and meta-analyses found OMA cystectomy not to lead to a significant decline in AFC [14,46]. Indeed, some studies suggest a rise in AFC post cystectomy [34,35,60,61]. Various studies have reported an advantage of ablative procedures over cystectomy regarding postoperative AFC [21,22,56,57,60,66]. A single retrospective study reported on ovarian reserve parameters after laparoscopic OMA sclerotherapy and linked this approach with a decline in both AMH and AFC [52].

Surgery for recurrent OMA may be a particularly challenging scenario. The deleterious effect of repeat OMA excision on the ovarian reserve in terms of recurrence has been demonstrated [140,141]. In the context of IVF/ICSI, second-line surgery for recurrent OMA leads to higher total dose and days of gonadotrophin use, and lower numbers of oocytes and grade 1 and 2 embryos, as well as lower embryo implantation rates and clinical pregnancy rates per cycle, compared to controls [142]. Kim et al. performed laparoscopic cystectomies for recurrent OMAs and identified a 20.8% recurrence rate and a 13.1% re-operation rate after the second cystectomy. Highers rASRM scores and disease stages were associated with higher risk of recurrence [143].

OMA per se negatively affects fertility, with an impact on ovarian [69,70,71] and, possibly, endometrial level [72,73]. Regarding the impact of unoperated OMA on MAR outcomes, studies have reported contradictory results on response to ovarian stimulation, oocyte and embryo quality, and clinical and livebirth rates [75,76,77,78,79,80,81,82,83,84,85,86]. Cystectomy has, traditionally, been considered superior to non-excisional approaches in terms of chances of spontaneous conception, supported by two RCTs comparing cystectomy to cyst coagulation using bipolar current [29,30,144,145]. No significant differences between surgical approaches, in terms of overall postoperative pregnancy rates, have been reported by any of the various studies [54,55,94,95,96]. Various systematic reviews and meta-analyses have demonstrated no significant improvement in clinical or livebirth rate by surgery pre MAR, compared to conservative management OMA using MAR only [17,97,98,99]. However, another study demonstrated a significant increase in the chances of success of infertility treatment following OMA surgery [69]. Albeit not statistically significant, the combination of surgery and ART may be the most successful in terms of livebirth rates [87].

The comparison of recurrence rates through various series is much more challenging, as most patients may, continuously or intermittently, use hormonal contraceptives postoperatively. The ovary represents a localization of the disease with a high postoperative recurrence rate in normo-ovulatory patients. A randomized study comparing patients undergoing OMA cystectomy followed by continuous oral contraceptive pill intake vs. cyclic oral contraceptive pill intake vs. no hormonal treatment clearly demonstrated that amenorrhea reduced the rate of postoperative OMA recurrence 3.5-fold [146]. It should be emphasized that 29% of women with complete excision of OMA and no postoperative hormonal treatment developed a recurrent OMA within 24 months after the surgery. This rate (which represents the pure likelihood of postoperative OMA recurrence) is higher than the majority of postoperative recurrence rates reported in various studies where postoperative pill intake is not clearly provided. Consequently, accurate comparison of recurrence rates following the use of various techniques should only include studies enrolling patients free of hormonal treatment during the whole follow-up period.

## 5. Conclusions

OMA per se exerts a negative effect on ovarian reserve and fertility. On the other hand, its surgical management may further aggravate the condition, due to inadvertent injury to ovarian parenchyma. Of the available surgical approaches, cystectomy appears advantageous in terms of reduced recurrence rates and probability of spontaneous conception when performed in women with no past history of ovarian surgeries. However, loss of normal ovarian tissue should be anticipated, even in experienced hands, causing, at least temporarily, a reduction in AMH. Furthermore, little information exists about fertility outcomes in women undergoing repeated cystectomies, where the loss of ovarian tissue may further increase. Alternative techniques, namely, ablative approaches, appear promising, as they have an ovarian tissue-sparing goal, thus causing less inadvertent injury to ovarian parenchyma. Checking AMH levels preoperatively may be useful in choosing the correct technique (as high-risk groups, such as patients with low AMH, may benefit from non-excisional techniques) and informing women of chances of postoperative conception. There appears to be no single technique that is clearly advantageous over the others as regards chances of postoperative pregnancy. Surgical management of OMA may significantly improve chances of conception. In the context of MAR, although surgery pre MAR has not demonstrated a clear benefit in terms of clinical pregnancy and livebirth rate, the combination of surgery followed by MAR may yield the optimal reproductive outcome. Individualization of care and optimal surgical technique are of paramount importance. Last but not least, it should be emphasized that, since surgical expertise may vary greatly between different units and clinicians, results may also vary accordingly.

## 6. Future Directions

In the future, more accurate information needs to be provided through randomized controlled trials comparing the available techniques, in terms of ovarian reserve parameters and reproductive outcomes, in patients free of postoperative hormonal treatment. We also need more original research on the topic of surgery or no surgery pre MAR in women with OMA. In women who will undergo surgery pre MAR, more research is needed to decide on the optimal surgical approach. In the future, the introduction of novel approaches and technologies that result in even less damage to the healthy ovarian parenchyma would be most welcome.

## Figures and Tables

**Table 1:** Published studies on ovarian endometrioma (OMA) cystectomy and reported outcomes. TP: Total pregnancy (number and percentage). SP: Spontaneous pregnancy (number and percentage). MAR: Medically assisted reproduction (pregnancies achieved through MAR, number and percentage). NS: Not specified. AMH: Anti-Müllerian hormone. AFC: Antral follicular count. Ng/mL: Nanograms/milliliter. Values are at the end of follow-up, unless otherwise specified.

Author, Year	Study Design	Mean Age (Years)	Number of Patients	Control Group	Follow-Up (Months)	Recurrence	TP	SP	MAR	AMH (ng/mL)	AFC
Celik et al., 2012 [34]	Prospective	28.4 ± 5.7	65	-	6	NS	10 (15.4%)	NS	NS	0.72 ± 0.79	6.4 ± 2.2
Taniguchi et al., 2016 [89]	Prospective	31.7 ± 5.4	40	Cystectomy for other benign ovarian cyst	18	NS	20 (50%)	10 (50%)	10 (50%)	NS	NS
Saito et al., 2018 [111]	Prospective	37 [27,28,29,30,31,32,33,34,35,36,37,38,39,40,41,42] (bilateral), 32 [21,22,23,24,25,26,27,28,29,30,31,32,33,34,35,36,37,38,39,40,41] (unilateral)	34 (10 bilateral, 24 unilateral)	Vaporization with bipolar current (Unilateral and bilateral)	12	0	NS	NS	NS	2.5 ± 1.7 (unilateral)/ 0.8 ± 0.7 (bilateral)	NS
Saito et al., 2014 [53]	Prospective	35.9 ± 6.2 (bilateral), 33.7 ± 6.0 (unilateral)	68 (28 bilateral, 40 unilateral)	Vaporization with bipolar current (Unilateral and bilateral)	1	NS	NS	NS	NS	1.7 ± 1.7 (unilateral)/0.5 ± 0.4 (bilateral)	NS
Urman et al., 2013 [62]	Prospective	32.7 ± 6.1	25	-	6	0	3 (43%)	2/3 (67%)	1/3 (33%)	NS	NS
Sweed et al., 2019 [45]	Prospective, randomized	27.1 ± 4.6	61	Deroofing of ovarian endometrioma (OMA)	12	11 (20.4%)	NS	NS	NS	1.39 ± 0.76	3.17 ± 1.36
Alborzi et al., 2014 [35]	Prospective	28.43 ± 5.35	193 (72 bilateral, 121 unilateral)	-	9	NS	NS	NS	NS	1.77 ± 1.76 (at 9 months)	10.75 ± 3.68 (at 3 months)
Sugita et al., 2013 [39]	Prospective	34.6 ± 5.5 (bilateral), 34.0 ± 4.5 (unilateral)	39 (22 unilateral, 17 bilateral)	Laparoscopic Myomectomy	12	NS	NS	NS	NS	2.10 [0.85, 3.48]	NS
Lee et al., 2011 [36]	Prospective	29.9 ± 4.5	13	-	3	NS	NS	NS	NS	3.29 ± 2.11	NS
Biacchiardi et al., 2011 [37]	Prospective	34.2 ± 5.4	43	-	9	NS	NS	NS	NS	1.3 ± 0.3	5.1 ± 3.6
Iwase et al., 2010 [112]	Prospective	33.3 ± 5.0	29 (16 unilateral, 13 bilateral)	Non-endometriotic ovarian cysts, fibroids	1	NS	NS	NS	NS	Median: 2.24, Range: 0.11–7.15	NS
Var et al., 2011 [67]	Prospective	27.04 ± 3.90	48	Coagulation with bipolar current	6	0	NS	NS	NS	NS	3.67 ± 1.26
Hwu et al., 2011 [113]	Retrospective	33.27 ± 4.09	31	Infertile patients without endometrioma	3	NS	NS	NS	NS	2.01 ± 0.21	NS
Shao et al., 2016 [114]	Prospective	29.1 (21–35)	68 (36 unilateral, 32 bilateral)	-	12	NS	NS	NS	NS	4.07 ± 2.06 (unilateral), 2.26 ± 1.88 (bilateral)	NS
Kwon et al., 2014 [115]	Prospective	31.72 ± 5.71	68 (42 unilateral, 26 bilateral)	Non-endometriotic ovarian cysts	3	NS	NS	NS	NS	3.22 ± 2.09	NS
Ercan et al., 2011 [48]	Prospective	29.4 ± 4.6	36	-	3	NS	NS	NS	NS	1.95 ± 0.62	3.7 ± 2.1
Uncu et al., 2013 [10]	Prospective	29.0 ± 5.4	30	No ovarian cyst	6	NS	NS	NS	NS	2.8 ± 2.2	9.7 ± 4.8
Chen et al., 2014 [116]	Prospective	30.38 ± 5.13	40	Non-endometriotic ovarian cysts, tubal factor infertility	1	NS	NS	NS	NS	0.69 ± 0.89	NS
Ding et al., 2015 [42]	Prospective	32.67 ± 4.89 (bilateral), 31.97 ± 4.59 (unilateral)	50 (21 unilateral, 29 bilateral)	Laparoscopic myomectomy or laparoscopic hydrotubation and fimbrioplasty (but no ovarian surgery)	12	NS	NS	NS	NS	0.075 ± 0.04 (bilateral), 0.08 ± 0.06 (unilateral)	4.80 ± 1.32 (bilateral), 5.60 ± 1.31 (unilateral)
Salihoglu et al., 2016 [117]	Prospective	NS	34	Laparoscopic cystectomy for non-endometriotic ovarian cyst	2	NS	NS	NS	NS	2.5 ± 1.6	7 (3–12)
Kashi et al., 2016 [118]	Prospective	29.66 ± 5.56	70 (45 unilateral, 25 bilateral)	-	6	NS	NS	NS	NS	1.32 ± 0.16	NS
Goodman et al., 2016 [11]	Prospective	32.1 ± 5.6	58	Peritoneal endometriosis; no endometriosis	6	NS	NS	NS	NS	1.41 (0.97–1.85).	NS
Rawat et al., 2019 [54]	Prospective Randomized	23.5 ± 4.47	10	Laparoscopic cystectomy with “cutting and coagulation” of ovarian endometrioma at the hilum	12	NS	1 (10%)	1/1 (100%)	0	4.27 ± 1.02	6.6 ± 2.06
Litta et al., 2013 [119]	Prospective	31 ± 6	25	-	3	NS	NS	NS	NS	3.00 (1.27–4.08)	NS
Jang et al., 2014 [120]	Prospective	27.0 ± 4.3	12	Laparoscopic cystectomy for non-endometriotic ovarian cyst	3	NS	NS	NS	NS	2.19 (1.49–3.60)	10.50 (8.50–4.00)
Bhat et al., 2014 [61]	Prospective	29.2 ± 3.6	73	-	7	NS	NS	NS	NS	NS	4.1 ± 1.5
Tanprasertkul et al., 2014 [121]	Prospective	32.74 ± 6.98	39	Laparoscopic non-ovarian pelvic surgery	6	NS	NS	NS	NS	1.69 ± 1.63	NS
Georgievska et al., 2014 [60]	Prospective	31 ± 6	40	-	3	NS	NS	NS	NS	NS	5.48 ± 1.96
Georgievska et al., 2015 [122]	Prospective	30.83 ± 5.32	30	Drainage and bipolar coagulation of OMA cyst wall	3	NS	NS	NS	NS	NS	6.23 ± 1.57
Vignali et al., 2015 [44]	Prospective	33 ± 6.17	22	-	12	NS	NS	NS	NS	4.01 ± 3.39	NS
Aşıcıoğlu et al., 2018 [47]	Retrospective	30.1 ± 5.3	44	-	3	NS	NS	NS	NS	2.1 ± 0.3	NS
Kovačević et al., 2018 [43]	Prospective	30.3 ± 4.5	54 (37 unilateral, 17 bilateral)	-	12	NS	24 (77.4%)	18 (75%)	6 (25%)	1.72 ± 1.23 (unilateral), 0.89 ± 0.82 (bilateral)	NS
Karadağ et al., 2020 [123]	Prospective	30.13 ± 4.61	36	Laparoscopic cystectomy for ovarian dermoid cysts	3	NS	NS	NS	NS	1.47 ± 0.55	2.16 ± 0.94
Chen et al., 2021 [55]	Retrospective	28.65 ± 3.66	46	Drainage and bipolar coagulation of OMA cyst wall	30.40 ± 3.83	0	27 (71.05%)	16 (59%)	11 (41%)	3.40 ± 1.35	NS
Anh et al., 2022 [38]	Prospective	28.5 (25–34)	104 (77 unilateral, 27 bilateral)	-	12	NS	NS	19	NS	2.39 (1.44–3.87) (unilateral), 0.92 (0.32–1.23) (bilateral)	NS

**Table 2:** Published studies on ovarian endometrioma (OMA) ablation by laser and reported outcomes. KTP: Potassium–Titanyl–Phosphate. CO_2_: Carbon dioxide. TP: Total pregnancy (number and percentage, where available). SP: Spontaneous pregnancy (number and percentage, where available). MAR: Medically assisted reproduction (pregnancies achieved through MAR, number and percentage, where available). NS: Not specified. AMH: Anti-Müllerian hormone. AFC: Antral follicular count. Ng/mL: Nanograms/milliliter. Values are at the end of follow-up, unless otherwise specified.

Author, Year	Study Design	Mean Age (Years)	Number of Patients	Control Group	Laser Type	Follow-Up (Months)	Recurrence	TP	SP	MAR	AMH (ng/mL)	AFC
Shimizu et al., 2010 [124]	Retrospective	30.8 ± 3.3	45	-	KTP	46	11 (24.4%)	34 (75.6%)	22 (64.7%)	12 (35.3%)	NS	NS
Carmona et al., 2011 [65]	Prospective randomized	32.3 ± 5.9	38	Cystectomy	CO_2_	60	14 (37%)	11 (44.4%)	8 (72.7%)	3 (27.3%)	NS	5.4 ± 2.0
Candiani et al., 2018 [56]	Prospective randomized	32.1 ± 4.8	30	Cystectomy	CO_2_	3	0	3 (25%)	3 (100%)	NS	1.9 ± 0.9	8.6 ± 4.2
Ottolina et al., 2017 [63]	Prospective	32.9 ± 5.7	15	-	CO_2_	3	0	1 (16.7%)	1 (100%)	ΝS	NS	8.1
Gheit et al., 2014 [57]	Prospective	27.4 ± 4.5	40	Cystectomy	Argon Beam	2 weeks	NS	NS	NS	NS	2.65 ± 1.38	7.6 ± 1.9
Pados et al., 2010 [21]	Prospective randomized	29.9 ± 1.8	10	Cystectomy	CO_2_ (“3-step procedure”)	12	2 (20%)	NS	NS	NS	NS	4.36 ± 0.8
Tsolakidis et al., 2010 [58]	Prospective randomized	29.9 ± 1.8	10	Cystectomy	CO_2_ (“3-step procedure”)	12	2 (20%)	NS	NS	NS	3.99 ± 0.6	4.36 ± 0.8
Rius et al., 2020 [66]	Prospective randomized	32.13 ± 6.56	16	Cystectomy	CO_2_	6	0	3 (33.3%)	NS	NS	NS	9.33 ± 6.2

**Table 3:** Published studies on ovarian endometrioma (OMA) ablation by plasma energy and reported outcomes. TP: Total pregnancy (number and percentage). SP: Spontaneous pregnancy (number and percentage). MAR: Medically assisted reproduction (pregnancies achieved through MAR, number and percentage). NS: Not specified. AMH: Anti-Müllerian hormone. AFC: Antral follicular count. Ng/mL: Nanograms/milliliter. Values are at the end of follow-up, unless otherwise specified.

Author, Year	Study Design	Mean Age (Years)	Number of Patients	Control Group	Follow-Up (Months)	Recurrence	TP	SP	MAR	AMH (ng/mL)	AFC
Roman et al., 2013 [90]	Retrospective	32 ± 4.85	55	-	20.6 ± 7.2	6 (10.9%)	22 (67%)	13 (59%)	9 (40.9%)	NS	NS
Motte et al., 2016 [103]	Retrospective	30.9 ± 4.4	37	No endometriosis	NS	NS	25 (67%)	3 (12%)	22 (88%)	NS	NS
Auber et al., 2011 [64]	Retrospective	32.4 ± 6.2	10	-	3	NS	NS	NS	NS	NS	18% decrease compared to contralateral healthy ovary
Roman et al., 2011 [125]	Retrospective	31.6 ± 5.2	15	Cystectomy	3	NS	NS	NS	NS	NS	5.5 ± 3.9
Roman et al., 2014 [51]	Prospective	30.6 ± 4.8	22	-	18.2 ± 8	1 (5%)	8 (73%)	3 (37%)	5 (63%)	3.1 ± 2.2	NS
Roman et al., 2015 [91]	Prospective	31.4 ± 5.1	124	-	32 ± 18	18 (14.5%)	51 (61.4%)	33 (64.7%)	18 (35.3%)	NS	NS
Mircea et al., 2016 [95]	Prospective	31 ± 4.3	64	Cystectomy	35.3 ± 17.5	NS	44 (68.7%)	18 (40.9%)	26 (59.1%)	NS	NS
Lockyer et al., 2019 [92]	Retrospective	31.8 ± 5.9	21	-	10 (3 to 31)	2 (9.5%)	6 (46.2%)	0	6 (100%)	NS	NS
Puscasiu et al., 2023 [96]	Retrospective (three-arm)	30.6 ± 4.5	204	i. Cystectomy ii. Drainage	50 ± 26 (12 to 120)	NS	132 (65%)	57 (43%)	75 (57%)	NS	NS

**Table 4:** Published studies on ovarian endometrioma (OMA) managed by combined procedures and reported outcomes. TP: Total pregnancy (number and percentage). SP: Spontaneous pregnancy (number and percentage). MAR: Medically assisted reproduction (pregnancies achieved through MAR, number and percentage). NS: Not specified. AMH: Anti-Müllerian hormone. AFC: Antral follicular count. Ng/mL: Nanograms/milliliter. Values are at the end of follow-up, unless otherwise specified.

Author, Year	Study Design	Mean Age (Years)	Number of Patients	Control Group	Type of Combined Procedure	Length of Follow-Up (Months)	Recurrence	TP	SP	MAR	AMH (ng/mL)	AFC
Donnez et al., 2010 [25]	Prospective	29.2 ± 3.7	52	-	Partial Cystectomy and CO_2_ Laser ablation (close to hilum)	6	1 (2%)	15 (41%)	NS	NS	NS	6.1 ± 3.2
Supermaniam, 2021 [27]	Retrospective	31.9	143	-	Vasopressin injection, partial cystectomy, bipolar coagulation (close to hilum), ovarian reconstruction with suturing	NS	NS	38 (50%)	32 (84%)	6 (16%)	NS	NS
Muzii, 2016 [68]	Prospective Randomized	32.9 ± 5.7	51	Cystectomy	Partial Cystectomy and bipolar coagulation (close to hilum)	6	1 (2%)	NS	NS	NS	NS	4.4 ± 2.3

**Table 5:** Published studies on ovarian endometrioma (OMA) managed by laparoscopic ethanol sclerotherapy and reported outcomes. TP: Total pregnancy (number and percentage). SP: Spontaneous pregnancy (number and percentage). MAR: Medically assisted reproduction (pregnancies achieved through MAR, number and percentage). NS: Not specified. AMH: Anti-Müllerian hormone. AFC: Antral follicular count. Ng/mL: Nanograms/milliliter. Values are at the end of follow-up, unless otherwise specified.

Author, Year	Study Design	Mean Age (Years)	Number of Patients	Control Group	Follow-Up (Months)	Recurrence	TP	SP	MAR	AMH (ng/mL)	AFC
Crestani et al., 2023 [52]	Retrospective	33.2 ± 4.7	69	-	17.5 ± 4.6	6 (11.8%)	18 (40.1%)	7 (39%)	11 (61%)	2 ± 1.7	12 ± 9.6
De Cicco Nardone, 2020 [93]	Retrospective	32 (19–40)	53	-	31 (12–60)	5 (9%)	16 (57%)	14 (87%)	2 (13%)	NS	NS

## Data Availability

Not applicable.

## References

[1] Moen M.H. (1987). Endometriosis in women at interval sterilization. Acta Obstet. Gynecol. Scand..

[2] Nisolle M., Donnez J. (1997). Peritoneal endometriosis, ovarian endometriosis, and adenomyotic nodules of the rectovaginal septum are three different entities. Fertil. Steril..

[3] Busacca M., Vignali M. (2003). Ovarian endometriosis: From pathogenesis to surgical treatment. Curr. Opin. Obstet. Gynecol..

[4] Fleming R., Seifer D.B., Frattarelli J.L., Ruman J. (2015). Assessing ovarian response: Antral follicle count versus anti-Müllerian hormone. Reprod. Biomed. Online.

[5] Ubaldi F., Vaiarelli A., D’Anna R., Rienzi L. (2014). Management of poor responders in IVF: Is there anything new?. BioMed Res. Int..

[6] Streuli I., de Ziegler D., Gayet V., Santulli P., Bijaoui G., de Mouzon J., Chapron C. (2012). In women with endometriosis anti-Mullerian hormone levels are decreased only in those with previous endometrioma surgery. Hum. Reprod..

[7] Raffi F., Metwally M., Amer S. (2012). The Impact of Excision of Ovarian Endometrioma on Ovarian Reserve: A Systematic Review and Meta-Analysis. J. Clin. Endocrinol. Metab..

[8] Rzeszowska M., Leszcz A., Putowski L., Hałabiś M., Tkaczuk-Włach J., Kotarski J., Polak G. (2016). Anti-Müllerian hormone: Structure, properties and appliance. Ginekol. Polska.

[9] Muzii L., Di Tucci C., Di Feliciantonio M., Galati G., Di Donato V., Musella A., Palaia I., Panici P.B. (2018). Antimüllerian hormone is reduced in the presence of ovarian endometriomas: A systematic review and meta-analysis. Fertil. Steril..

[10] Uncu G., Kasapoglu I., Ozerkan K., Seyhan A., Yilmaztepe A.O., Ata B. (2013). Prospective assessment of the impact of endometriomas and their removal on ovarian reserve and determinants of the rate of decline in ovarian reserve. Hum. Reprod..

[11] Goodman L.R., Goldberg J.M., Flyckt R.L., Gupta M., Harwalker J., Falcone T. (2016). Effect of surgery on ovarian reserve in women with endometriomas, endometriosis and controls. Am. J. Obstet. Gynecol..

[12] Kasapoglu I., Ata B., Uyaniklar O., Seyhan A., Orhan A., Oguz S.Y., Uncu G. (2018). Endometrioma-related reduction in ovarian reserve (ERROR): A prospective longitudinal study. Fertil. Steril..

[13] Nieweglowska D., Hajdyla-Banas I., Pitynski K., Banas T., Grabowska O., Juszczyk G., Ludwin A., Jach R. (2015). Age-related trends in anti-Mullerian hormone serum level in women with unilateral and bilateral ovarian endometriomas prior to surgery. Reprod. Biol. Endocrinol..

[14] Muzii L., Di Tucci C., Di Feliciantonio M., Marchetti C., Perniola G., Panici P.B. (2014). The effect of surgery for endometrioma on ovarian reserve evaluated by antral follicle count: A systematic review and meta-analysis. Hum. Reprod..

[15] Halis G., Arici A. (2004). Endometriosis and Inflammation in Infertility. Ann. N. Y. Acad. Sci..

[16] Martins W.P. (2013). Questionable value of absolute mean gray value for clinical practice. Ultrasound Obstet. Gynecol..

[17] Hamdan M., Dunselman G., Li T., Cheong Y. (2015). The impact of endometrioma on IVF/ICSI outcomes: A systematic review and meta-analysis. Hum. Reprod. Updat..

[18] Lima M.L., Martins W.P., Neto M.A.C., Nastri C.O., Ferriani R.A., Navarro P.A. (2015). Assessment of ovarian reserve by antral follicle count in ovaries with endometrioma. Ultrasound Obstet. Gynecol..

[19] Inal Z.O., Ustun Y.E., Yilmaz N., Aktulay A., Bardakci Y., Gulerman C. (2019). Does the anti-Müllerian hormone truly reflect ovarian response in women with endometrioma?. J. Obstet. Gynaecol..

[20] Muzii L., Bianchi A., Bellati F., Cristi E., Pernice M., Zullo M.A., Angioli R., Panici P.B. (2007). Histologic analysis of endometriomas: What the surgeon needs to know. Fertil. Steril..

[21] Pados G., Tsolakidis D., Assimakopoulos E., Athanatos D., Tarlatzis B. (2010). Sonographic changes after laparoscopic cystectomy compared with three-stage management in patients with ovarian endometriomas: A prospective randomized study. Hum. Reprod..

[22] Roman H., Pura I., Tarta O., Mokdad C., Auber M., Bourdel N., Marpeau L., Sabourin J.C. (2011). Vaporization of ovarian endometrioma using plasma energy: Histologic findings of a pilot study. Fertil. Steril..

[23] Deb S., Deen S., Ashford K.S., Harwood A., Newman C., Powell M.C. (2010). Histological quantification of the tissue damage caused by PlasmaJet™ coagulator. Gynecol. Surg..

[24] Donnez J., Nisolle M., Gillet N., Smets M., Bassil S., Casanas-Roux F. (1996). Large ovarian endometriomas. Hum. Reprod..

[25] Donnez J., Lousse J.-C., Jadoul P., Donnez O., Squifflet J. (2010). Laparoscopic management of endometriomas using a combined technique of excisional (cystectomy) and ablative surgery. Fertil. Steril..

[26] Unlu C., Yildirim G. (2014). Ovarian cystectomy in endometriomas: Combined approach. J. Turk. Gynecol. Assoc..

[27] Supermaniam S., Thye W.L. (2021). Laparoscopic cystectomy in treating women with endometrioma and pregnancy outcome—A case series. Med. J. Malays..

[28] Crestani A., Merlot B., Dennis T., Roman H. (2022). Laparoscopic sclerotherapy for an endometrioma in 10 steps. Fertil. Steril..

[29] Becker C.M., Bokor A., Heikinheimo O., Horne A., Jansen F., Kiesel L., King K., Kvaskoff M., Nap A., Petersen K. (2022). ESHRE guideline: Endometriosis. Hum. Reprod. Open.

[30] Hart R.J., Hickey M., Maouris P., Buckett W. (2008). Excisional surgery versus ablative surgery for ovarian endometriomata. Cochrane Database Syst. Rev..

[31] Exacoustos C., Zupi E., Amadio A., Szabolcs B., De Vivo B., Marconi D., Romanini M.E., Arduini D. (2004). Laparoscopic removal of endometriomas: Sonographic evaluation of residual functioning ovarian tissue. Am. J. Obstet. Gynecol..

[32] Busacca M., Riparini J., Somigliana E., Oggioni G., Izzo S., Vignali M., Candiani M. (2006). Postsurgical ovarian failure after laparoscopic excision of bilateral endometriomas. Am. J. Obstet. Gynecol..

[33] Somigliana E., Berlanda N., Benaglia L., Viganò P., Vercellini P., Fedele L. (2012). Surgical excision of endometriomas and ovarian reserve: A systematic review on serum antimüllerian hormone level modifications. Fertil. Steril..

[34] Celik H.G., Dogan E., Okyay E., Ulukus C., Saatli B., Uysal S., Koyuncuoglu M. (2012). Effect of laparoscopic excision of endometriomas on ovarian reserve: Serial changes in the serum antimüllerian hormone levels. Fertil. Steril..

[35] Alborzi S., Keramati P., Younesi M., Samsami A., Dadras N. (2014). The impact of laparoscopic cystectomy on ovarian reserve in patients with unilateral and bilateral endometriomas. Fertil. Steril..

[36] Lee D.-Y., Kim N.Y., Kim M.J., Yoon B.-K., Choi D. (2011). Effects of laparoscopic surgery on serum anti-müllerian hormone levels in reproductive-aged women with endometrioma. Gynecol. Endocrinol..

[37] Biacchiardi C.P., Piane L.D., Camanni M., Deltetto F., Delpiano E.M., Marchino G.L., Gennarelli G., Revelli A. (2011). Laparoscopic stripping of endometriomas negatively affects ovarian follicular reserve even if performed by experienced surgeons. Reprod. Biomed. Online.

[38] Anh N.D., Ha N.T.T., Tri N.M., Huynh D.K., Dat D.T., Thuong P.T.H., Toan N.K., Duc T.A., Hinh N.D., Van Tong H. (2022). Long-Term Follow-Up of Anti-Mullerian Hormone Levels after Laparoscopic Endometrioma Cystectomy. Int. J. Med. Sci..

[39] Sugita A., Iwase A., Goto M., Nakahara T., Nakamura T., Kondo M., Osuka S., Mori M., Saito A., Kikkawa F. (2013). One-year follow-up of serum antimüllerian hormone levels in patients with cystectomy: Are different sequential changes due to different mechanisms causing damage to the ovarian reserve?. Fertil. Steril..

[40] Wang Y., Ruan X., Lu D., Sheng J., Mueck A.O. (2019). Effect of laparoscopic endometrioma cystectomy on anti-Müllerian hormone (AMH) levels. Gynecol. Endocrinol..

[41] Kostrzewa M., Wilczyński J.R., Głowacka E., Żyła M., Szyłło K., Stachowiak G. (2019). One-year follow-up of ovarian reserve by three methods in women after laparoscopic cystectomy for endometrioma and benign ovarian cysts. Int. J. Gynecol. Obstet..

[42] Ding Y., Yuan Y., Ding J., Chen Y., Zhang X., Hua K. (2015). Comprehensive Assessment of the Impact of Laparoscopic Ovarian Cystectomy on Ovarian Reserve. J. Minim. Invasive Gynecol..

[43] Kovačević V.M., Anđelić L.M., Jovanović A.M. (2018). Changes in serum antimüllerian hormone levels in patients 6 and 12 months after endometrioma stripping surgery. Fertil. Steril..

[44] Vignali M., Mabrouk M., Ciocca E., Alabiso G., di Prun A.B., Gentilini D., Busacca M. (2015). Surgical excision of ovarian endometriomas: Does it truly impair ovarian reserve? Long term anti-Müllerian hormone (AMH) changes after surgery. J. Obstet. Gynaecol. Res..

[45] Sweed M.S., Makled A.K., El-Sayed M.A., Shawky M.E., Abd-Elhady H.A., Mansour A.M., Mohamed R.M., Hemeda H., Nasr-Eldin E.A., Attia N.S. (2019). Ovarian Reserve Following Laparoscopic Ovarian Cystectomy vs Cyst Deroofing for Endometriomas. J. Minim. Invasive Gynecol..

[46] Younis J.S., Shapso N., Fleming R., Ben-Shlomo I., Izhaki I. (2019). Impact of unilateral versus bilateral ovarian endometriotic cystectomy on ovarian reserve: A systematic review and meta-analysis. Hum. Reprod. Updat..

[47] Aşıcıoğlu O., Temizkan O., Arıcı B., Aşıcıoğlu B.B. (2018). Clinical Characteristics and Outcomes of Laparoscopic Surgery in Ovarian Endometrioma Cases Treated at a Gynecology Clinic. Şişli Etfal Hastanesi Tip Bülteni.

[48] Ercan C.M., Duru N.K., Karasahin K.E., Coksuer H., Dede M., Baser I. (2011). Ultrasonographic evaluation and anti-mullerian hormone levels after laparoscopic stripping of unilateral endometriomas. Eur. J. Obstet. Gynecol. Reprod. Biol..

[49] Kitajima M., Matsumoto K., Murakami N., Harada A., Kitajima Y., Masuzaki H., Miura K. (2020). Ovarian reserve after three-step laparoscopic surgery for endometriomas utilizing dienogest: A pilot study. Reprod. Med. Biol..

[50] Muraoka A., Osuka S., Yabuki A., Bayasula, Yoshihara M., Tanaka H., Sonehara R., Miyake N., Murakami M., Yoshita S. (2021). Impact of perioperative use of GnRH agonist or dienogest on ovarian reserve after cystectomy for endometriomas: A randomized controlled trial. Reprod. Biol. Endocrinol..

[51] Roman H., Bubenheim M., Auber M., Marpeau L., Puscasiu L. (2014). Antimullerian Hormone Level and Endometrioma Ablation Using Plasma Energy. JSLS J. Soc. Laparosc. Robot. Surg..

[52] Crestani A., Merlot B., Dennis T., Chanavaz-Lacheray I., Roman H. (2022). Impact of Laparoscopic Sclerotherapy for Ovarian Endometriomas on Ovarian Reserve. J. Minim. Invasive Gynecol..

[53] Saito N., Okuda K., Yuguchi H., Yamashita Y., Terai Y., Ohmichi M. (2014). Compared with Cystectomy, Is Ovarian Vaporization of Endometriotic Cysts Truly More Effective in Maintaining Ovarian Reserve?. J. Minim. Invasive Gynecol..

[54] Rawat S., Roy K., Kumar S., Sharma J., Singh N., Chodsol K. (2019). A randomised comparative study of ovarian reserve following two different techniques of laparoscopic cystectomy in ovarian endometrioma. Int. J. Clin. Obstet. Gynaecol..

[55] Chen J., Huang D., Zhang J., Shi L., Li J., Zhang S. (2021). The effect of laparoscopic excisional and ablative surgery on ovarian reserve in patients with endometriomas: A retrospective study. Medicine.

[56] Candiani M., Ottolina J., Posadzka E., Ferrari S., Castellano L.M., Tandoi I., Pagliardini L., Nocuń A., Jach R. (2018). Assessment of ovarian reserve after cystectomy versus ‘one-step’ laser vaporization in the treatment of ovarian endometrioma: A small randomized clinical trial. Hum. Reprod..

[57] Gheit S.A., Hosny A.N., Yassin M.S., Gemei S., Shokry M.A.Z., Youssef M.A. (2014). Argon beam coagulator versus cystectomy for endometrioma treatment in infertile women and the impact on ovarian reserve. A case control study. Middle East Fertil. Soc. J..

[58] Tsolakidis D., Pados G., Vavilis D., Athanatos D., Tsalikis T., Giannakou A., Tarlatzis B.C. (2010). The impact on ovarian reserve after laparoscopic ovarian cystectomy versus three-stage management in patients with endometriomas: A prospective randomized study. Fertil. Steril..

[59] Giampaolino P., Bifulco G., Sardo A.D.S., Mercorio A., Bruzzese D., Di Carlo C. (2015). Endometrioma size is a relevant factor in selection of the most appropriate surgical technique: A prospective randomized preliminary study. Eur. J. Obstet. Gynecol. Reprod. Biol..

[60] Georgievska J., Sapunov S., Cekovska S., Vasilevska K. (2014). Ovarian Reserve after Laparoscopic Treatment of Unilateral Ovarian Endometrioma. Acta Inform. Medica.

[61] Bhat R.G., Dhulked S., Ramachandran A., Bhaktha R., Vasudeva A., Kumar P., Rao A.C.K. (2014). Laparoscopic cystectomy of endometrioma: Good surgical technique does not adversely affect ovarian reserve. J. Hum. Reprod. Sci..

[62] Urman B., Alper E., Yakin K., Oktem O., Aksoy S., Alatas C., Mercan R., Ata B. (2013). Removal of unilateral endometriomas is associated with immediate and sustained reduction in ovarian reserve. Reprod. Biomed. Online.

[63] Ottolina J., Castellano L.M., Ferrari S., Tandoi L., Candotti G., De Stefano F., Bartiromo L., Candiani M. (2017). The Impact on Ovarian Reserve of CO_2_ Laser Fiber Vaporization in the Treatment of Ovarian Endometrioma: A Prospective Clinical Trial. J. Endometr. Pelvic Pain Disord..

[64] Auber M., Bourdel N., Mokdad C., Martin C., Diguet A., Marpeau L., Roman H. (2011). Ultrasound ovarian assessments after endometrioma ablation using plasma energy. Fertil. Steril..

[65] Carmona F., Martínez-Zamora M.A., Rabanal A., Martínez-Román S., Balasch J. (2011). Ovarian cystectomy versus laser vaporization in the treatment of ovarian endometriomas: A randomized clinical trial with a five-year follow-up. Fertil. Steril..

[66] Rius M., Gracia M., Ros C., Martínez-Zamora M., Deguirior C., Quintas L., Carmona F. (2020). Impact of endometrioma surgery on ovarian reserve: A prospective, randomized, pilot study comparing stripping with CO_2_ laser vaporization in patients with bilateral endometriomas. J. Int. Med Res..

[67] Var T., Batioglu S., Tonguc E., Kahyaoglu I. (2011). The effect of laparoscopic ovarian cystectomy versus coagulation in bilateral endometriomas on ovarian reserve as determined by antral follicle count and ovarian volume: A prospective randomized study. Fertil. Steril..

[68] Muzii L., Achilli C., Bergamini V., Candiani M., Garavaglia E., Lazzeri L., Lecce F., Maiorana A., Maneschi F., Marana R. (2016). Comparison between the stripping technique and the combined excisional/ablative technique for the treatment of bilateral ovarian endometriomas: A multicentre RCT. Hum. Reprod..

[69] Raffi F., Amer S.A. (2014). Long-term reproductive performance after surgery for ovarian endometrioma. Eur. J. Obstet. Gynecol. Reprod. Biol..

[70] Benaglia L., Somigliana E., Vercellini P., Abbiati A., Ragni G., Fedele L. (2009). Endometriotic ovarian cysts negatively affect the rate of spontaneous ovulation. Hum. Reprod..

[71] Maneschi F., Marasá L., Incandela S., Mazzarese M., Zupi E. (1993). Ovarian cortex surrounding benign neoplasms: A histologic study. Am. J. Obstet. Gynecol..

[72] Celik O., Celik E., Turkcuoglu I., Yilmaz E., Ulas M., Simsek Y., Karaer A., Celik N., Aydin N.E., Ozerol I. (2013). Surgical Removal of Endometrioma Decreases the NF-kB1 (p50/105) and NF-kB p65 (Rel A) Expression in the Eutopic Endometrium during the Implantation Window. Reprod. Sci..

[73] Celik O., Unlu C., Otlu B., Celik N., Caliskan E. (2015). Laparoscopic endometrioma resection increases peri-implantation endometrial HOXA-10 and HOXA-11 mRNA expression. Fertil. Steril..

[74] Maggiore U.L.R., Scala C., Tafi E., Racca A., Biscaldi E., Vellone V.G., Venturini P.L., Ferrero S. (2017). Spontaneous fertility after expectant or surgical management of rectovaginal endometriosis in women with or without ovarian endometrioma: A retrospective analysis. Fertil. Steril..

[75] Gupta S., Agarwal A., Agarwal R., de Mola J.R.L. (2006). Impact of ovarian endometrioma on assisted reproduction outcomes. Reprod. Biomed. Online.

[76] Yang C., Geng Y., Li Y., Chen C., Gao Y. (2015). Impact of ovarian endometrioma on ovarian responsiveness and IVF: A systematic review and meta-analysis. Reprod. Biomed. Online.

[77] Somigliana E., Infantino M., Benedetti F., Arnoldi M., Calanna G., Ragni G. (2006). The presence of ovarian endometriomas is associated with a reduced responsiveness to gonadotropins. Fertil. Steril..

[78] Almog B., Shehata F., Sheizaf B., Tulandi T. (2010). Effect of different types of ovarian cyst on antral follicle count. Fertil. Steril..

[79] Esinler I., Bozdag G., Arikan I., Demir B., Yarali H. (2012). Endometrioma ≤3 cm in Diameter per se Does Not Affect Ovarian Reserve in Intracytoplasmic Sperm Injection Cycles. Gynecol. Obstet. Investig..

[80] Filippi F., Benaglia L., Paffoni A., Restelli L., Vercellini P., Somigliana E., Fedele L. (2014). Ovarian endometriomas and oocyte quality: Insights from in vitro fertilization cycles. Fertil. Steril..

[81] Deng Y., Ou Z., Yin M., Chen Z., Chen S., Sun L. (2022). Does current ovarian endometrioma increase the time for DOR patients to reach live birth in IVF?. BMC Pregnancy Childbirth.

[82] Dong X., Wang R., Zheng Y., Xiong T., Liao X., Huang B., Zhang H. (2014). Surgical treatment for endometrioma does not increase clinical pregnancy rate or live birth/ongoing pregnancy rate after fresh IVF/ICSI treatment. Am. J. Transl. Res..

[83] Wu Y., Yang R., Lan J., Lin H., Jiao X., Zhang Q. (2021). Ovarian Endometrioma Negatively Impacts Oocyte Quality and Quantity But Not Pregnancy Outcomes in Women Undergoing IVF/ICSI Treatment: A Retrospective Cohort Study. Front. Endocrinol..

[84] Liang Y., Yang X., Lan Y., Lei L., Li Y., Wang S. (2019). Effect of Endometrioma cystectomy on cytokines of follicular fluid and IVF outcomes. J. Ovarian Res..

[85] Opøien H.K., Fedorcsak P., Omland A.K., Åbyholm T., Bjercke S., Ertzeid G., Oldereid N., Mellembakken J.R., Tanbo T. (2012). In vitro fertilization is a successful treatment in endometriosis-associated infertility. Fertil. Steril..

[86] Zhou F., Zhao F., Jin X., Li C., Zhang S. (2022). Factors affecting clinical outcomes after IVF-ET for infertile young patients with ovarian endometrioma: A 5-year retrospective cohort study. Medicine.

[87] Alborzi S., Sorouri Z.Z., Askari E., Poordast T., Chamanara K. (2019). The success of various endometrioma treatments in infertility: A systematic review and meta-analysis of prospective studies. Reprod. Med. Biol..

[88] Harada M., Takahashi N., Hirata T., Koga K., Fujii T., Osuga Y. (2015). Laparoscopic excision of ovarian endometrioma does not exert a qualitative effect on ovarian function: Insights from in vitro fertilization and single embryo transfer cycles. J. Assist. Reprod. Genet..

[89] Taniguchi F., Sakamoto Y., Yabuta Y., Azuma Y., Hirakawa E., Nagira K., Uegaki T., Deura I., Hata K., Harada T. (2016). Analysis of pregnancy outcome and decline of anti-Müllerian hormone after laparoscopic cystectomy for ovarian endometriomas. J. Obstet. Gynaecol. Res..

[90] Roman H., Auber M., Bourdel N., Martin C., Marpeau L., Puscasiu L. (2013). Postoperative Recurrence and Fertility after Endometrioma Ablation Using Plasma Energy: Retrospective Assessment of a 3-Year Experience. J. Minim. Invasive Gynecol..

[91] Roman H., Quibel S., Auber M., Muszynski H., Huet E., Marpeau L., Tuech J.J. (2015). Recurrences and fertility after endometrioma ablation in women with and without colorectal endometriosis: A prospective cohort study. Hum. Reprod..

[92] Lockyer E.K., Schreurs A., Lier M., Dekker J., Melgers I., Mijatovic V. (2019). Treatment of ovarian endometriomas using plasma energy in endometriosis surgery: Effect on pelvic pain, return to work, pregnancy and cyst recurrence. Facts Views Vis. ObGyn.

[93] Nardone A.D.C., Carfagna P., Nardone C.D.C., Scambia G., Marana R., Nardone F.D.C. (2020). Laparoscopic Ethanol Sclerotherapy for Ovarian Endometriomas: Preliminary Results. J. Minim. Invasive Gynecol..

[94] Dan H., Limin F. (2013). Laparoscopic Ovarian Cystectomy versus Fenestration/Coagulation or Laser Vaporization for the Treatment of Endometriomas: A Meta-Analysis of Randomized Controlled Trials. Gynecol. Obstet. Investig..

[95] Mircea O., Puscasiu L., Resch B., Lucas J., Collinet P., von Theobald P., Merviel P., Roman H. (2016). Fertility Outcomes after Ablation Using Plasma Energy Versus Cystectomy in Infertile Women with Ovarian Endometrioma: A Multicentric Comparative Study. J. Minim. Invasive Gynecol..

[96] Puscasiu L., Mircea O., Hennetier C., Rubod C., Schmied R., Resch B., Merlot B., Roman H. (2022). Pregnancy rate following endometriomas management by ablation using plasma energy, cystectomy and drainage: A three-arm comparative study. Int. J. Gynecol. Obstet..

[97] Laursen J.B., Schroll J.B., Macklon K.T., Rudnicki M. (2017). Surgery versus conservative management of endometriomas in subfertile women. A systematic review. Acta Obstet. Gynecol. Scand..

[98] Nickkho-Amiry M., Savant R., Majumder K., Edi-O’sagie E., Akhtar M. (2018). The effect of surgical management of endometrioma on the IVF/ICSI outcomes when compared with no treatment? A systematic review and meta-analysis. Arch. Gynecol. Obstet..

[99] Tao X., Chen L., Ge S., Cai L. (2017). Weigh the pros and cons to ovarian reserve before stripping ovarian endometriomas prior to IVF/ICSI: A meta-analysis. PLoS ONE.

[100] Hosseinimousa S., Safdarian L., Aleyasin A., Aghahosseini M., Talebian M. (2022). Can Laparoscopic Cystectomy Improve Pregnancy Outcomes in Endometrioma? A Prospective Clinical Trial Study. Int. J. Fertil. Steril..

[101] Liu W., Sha T., Huang Y., Guo Z., Yan L., Ma J. (2021). Factors Influencing the Live Birth Rate Following Fresh Embryo Transfer Cycles in Infertile Women after Endometrioma Cystectomy. Front. Med..

[102] Tang Y., Chen S.-L., Chen X., He Y.-X., Ye D.-S., Guo W., Zheng H.-Y., Yang X.-H. (2013). Ovarian damage after laparoscopic endometrioma excision might be related to the size of cyst. Fertil. Steril..

[103] Motte I., Roman H., Clavier B., Jumeau F., Chanavaz-Lacheray I., Letailleur M., Darwish B., Rives N. (2016). In vitro fertilization outcomes after ablation of endometriomas using plasma energy: A retrospective case-control study. Gynecol. Obstet. Fertil..

[104] Benaglia L., Somigliana E., Vighi V., Ragni G., Vercellini P., Fedele L. (2010). Rate of severe ovarian damage following surgery for endometriomas. Hum. Reprod..

[105] Somigliana E., Benaglia L., Vercellini P., Paffoni A., Ragni G., Fedele L. (2011). Recurrent endometrioma and ovarian reserve: Biological connection or surgical paradox?. Am. J. Obstet. Gynecol..

[106] Roustan A., Perrin J., Debals-Gonthier M., Paulmyer-Lacroix O., Agostini A., Courbiere B. (2015). Surgical diminished ovarian reserve after endometrioma cystectomy versus idiopathic DOR: Comparison of in vitro fertilization outcome. Hum. Reprod..

[107] Hong S.B., Lee N.R., Kim S.K., Kim H., Jee B.C., Suh C.S., Kim S.H., Choi Y.M. (2017). In vitro fertilization outcomes in women with surgery induced diminished ovarian reserve after endometrioma operation: Comparison with diminished ovarian reserve without ovarian surgery. Obstet. Gynecol. Sci..

[108] Yu H.-T., Huang H.-Y., Soong Y.-K., Lee C.-L., Chao A., Wang C.-J. (2010). Laparoscopic ovarian cystectomy of endometriomas: Surgeons’ experience may affect ovarian reserve and live-born rate in infertile patients with in vitro fertilization-intracytoplasmic sperm injection. Eur. J. Obstet. Gynecol. Reprod. Biol..

[109] Yu H.-T., Huang H.-Y., Lee C.-L., Soong Y.-K., Wang C.-J. (2015). Side of ovarian endometrioma does not affect the outcome of in vitro fertilization/intracytoplasmic sperm injection in infertile women after laparoscopic cystectomy. J. Obstet. Gynaecol. Res..

[110] Yu H.-T., Huang H.-Y., Tseng H.-J., Wang C.-J., Lee C.-L., Soong Y.-K. (2017). Bilaterality of ovarian endometriomas does not affect the outcome of in vitro fertilization/intracytoplasmic sperm injection in infertile women after laparoscopic cystectomy. Biomed. J..

[111] Saito N., Yamashita Y., Okuda K., Kokunai K., Terai Y., Ohmichi M. (2018). Comparison of the impact of laparoscopic endometriotic cystectomy and vaporization on postoperative serum anti-Mullerian hormone levels. Asian J. Endosc. Surg..

[112] Iwase A., Hirokawa W., Goto M., Takikawa S., Nagatomo Y., Nakahara T., Manabe S., Kikkawa F. (2010). Serum anti-Müllerian hormone level is a useful marker for evaluating the impact of laparoscopic cystectomy on ovarian reserve. Fertil. Steril..

[113] Hwu Y.-M., Wu F.S.-Y., Li S.-H., Sun F.-J., Lin M.-H., Lee R.K.-K. (2011). The impact of endometrioma and laparoscopic cystectomy on serum anti-Müllerian hormone levels. Reprod. Biol. Endocrinol..

[114] Shao M.-J., Hu M., He Y.-Q., Xu X.-J. (2016). AMH trend after laparoscopic cystectomy and ovarian suturing in patients with endometriomas. Arch. Gynecol. Obstet..

[115] Kwon S.K., Kim S.H., Yun S.-C., Kim D.Y., Chae H.D., Kim C.-H., Kang B.M. (2014). Decline of serum antimüllerian hormone levels after laparoscopic ovarian cystectomy in endometrioma and other benign cysts: A prospective cohort study. Fertil. Steril..

[116] Chen Y., Pei H., Chang Y., Chen M., Wang H., Xie H., Yao S. (2014). The impact of endometrioma and laparoscopic cystectomy on ovarian reserve and the exploration of related factors assessed by serum anti-Mullerian hormone: A prospective cohort study. J. Ovarian Res..

[117] Salihoğlu K.N., Dilbaz B., Cırık D.A., Ozelci R., Ozkaya E., Mollamahmutoğlu L. (2016). Short-Term Impact of Laparoscopic Cystectomy on Ovarian Reserve Tests in Bilateral and Unilateral Endometriotic and Nonendometriotic Cysts. J. Minim. Invasive Gynecol..

[118] Kashi A.M., Chaichian S., Ariana S., Fazaeli M., Moradi Y., Rashidi M., Najmi Z. (2016). The impact of laparoscopic cystectomy on ovarian reserve in patients with unilateral and bilateral endometrioma. Int. J. Gynecol. Obstet..

[119] Litta P., D’agostino G., Conte L., Saccardi C., Cela V., Angioni S., Plebani M. (2013). Anti-Müllerian hormone trend after laparoscopic surgery in women with ovarian endometrioma. Gynecol. Endocrinol..

[120] Jang W.K., Lim S.Y., Park J.C., Lee K.R., Lee A., Rhee J.H. (2014). Surgical impact on serum anti-Müllerian hormone in women with benign ovarian cyst: A prospective study. Obstet. Gynecol. Sci..

[121] Tanprasertkul C., Manusook S., Somprasit C., Ekarattanawong S., Sreshthaputra O., Vutyavanich T. (2014). Antimullerian Hormone Changes after Laparoscopic Ovarian Cystectomy for Endometrioma Compared with the Nonovarian Conditions. Minim. Invasive Surg..

[122] Georgievska J., Sapunov S., Cekovska S., Vasilevska K. (2015). Effect of Two Laparoscopic Techniques for Treatment of Ovarian Endometrioma on Ovarian Reserve. Med Arch..

[123] Karadağ C., Demircan S., Turgut A., Çalışkan E. (2020). Effects of laparoscopic cystectomy on ovarian reserve in patients with endometrioma and dermoid cyst. J. Turk. Soc. Obstet. Gynecol..

[124] Shimizu Y., Takashima A., Takahashi K., Kita N., Fujiwara M., Murakami T. (2010). Long-term outcome, including pregnancy rate, recurrence rate and ovarian reserve, after laparoscopic laser ablation surgery in infertile women with endometrioma. J. Obstet. Gynaecol. Res..

[125] Roman H., Auber M., Mokdad C., Martin C., Diguet A., Marpeau L., Bourdel N. (2011). Ovarian endometrioma ablation using plasma energy versus cystectomy: A step toward better preservation of the ovarian parenchyma in women wishing to conceive. Fertil. Steril..

[126] Ercan C.M., Sakinci M., Duru N.K., Alanbay I., Karasahin K.E., Baser I. (2010). Antimullerian hormone levels after laparoscopic endometrioma stripping surgery. Gynecol. Endocrinol..

[127] Alborzi S., Foroughinia L., Kumar P.V., Asadi N., Alborzi S. (2009). A comparison of histopathologic findings of ovarian tissue inadvertently excised with endometrioma and other kinds of benign ovarian cyst in patients undergoing laparoscopy versus laparotomy. Fertil. Steril..

[128] Ata B., Turkgeldi E., Seyhan A., Urman B. (2015). Effect of Hemostatic Method on Ovarian Reserve Following Laparoscopic Endometrioma Excision; Comparison of Suture, Hemostatic Sealant, and Bipolar Dessication. A Systematic Review and Meta-Analysis. J. Minim. Invasive Gynecol..

[129] Deckers P., Ribeiro S.C., Simões R.d.S., Miyahara C.B.d.F., Baracat E.C. (2017). Systematic review and meta-analysis of the effect of bipolar electrocoagulation during laparoscopic ovarian endometrioma stripping on ovarian reserve. Int. J. Gynecol. Obstet..

[130] Ding W., Li M., Teng Y. (2015). The impact on ovarian reserve of haemostasis by bipolar coagulation versus suture following surgical stripping of ovarian endometrioma: A meta-analysis. Reprod. Biomed. Online.

[131] Roman H., Tarta O., Pura I., Opris I., Bourdel N., Marpeau L., Sabourin J.-C. (2010). Direct proportional relationship between endometrioma size and ovarian parenchyma inadvertently removed during cystectomy, and its implication on the management of enlarged endometriomas. Hum. Reprod..

[132] Lee J., Kang J., Lee H.J. (2022). Effect of Surgical Findings on Prediction of Postoperative Ovarian Reserve in Patients with Ovarian Endometrioma. Int. J. Women’s Heal..

[133] Hirokawa W., Iwase A., Goto M., Takikawa S., Nagatomo Y., Nakahara T., Bayasula B., Nakamura T., Manabe S., Kikkawa F. (2011). The post-operative decline in serum anti-Mullerian hormone correlates with the bilaterality and severity of endometriosis. Hum. Reprod..

[134] Romualdi D., Zannoni G.F., Lanzone A., Selvaggi L., Tagliaferri V., Vellone V.G., Campagna G., Guido M. (2011). Follicular loss in endoscopic surgery for ovarian endometriosis: Quantitative and qualitative observations. Fertil. Steril..

[135] Oh H.-K., Sin J.-I., Kim J.-H., Hong S.-Y., Lee T.-S., Choi Y.-S. (2011). Effect of age and stage of endometriosis on ovarian follicular loss during laparoscopic cystectomy for endometrioma. Int. J. Gynecol. Obstet..

[136] Dong Z., An J., Xie X., Wang Z., Sun P. (2019). Preoperative serum anti-Müllerian hormone level is a potential predictor of ovarian endometrioma severity and postoperative fertility. Eur. J. Obstet. Gynecol. Reprod. Biol..

[137] Zhou Y., Chen C., Hu C., Wang Y., Zhang X., Wu R. (2019). Predictive value of the serum anti-Müllerian level for spontaneous pregnancy in women after endometriosis surgery. J. Int. Med Res..

[138] Stochino-Loi E., Darwish B., Mircea O., Touleimat S., Millochau J.-C., Abo C., Angioni S., Roman H. (2017). Does preoperative antimüllerian hormone level influence postoperative pregnancy rate in women undergoing surgery for severe endometriosis?. Fertil. Steril..

[139] Iwase A., Nakamura T., Kato N., Goto M., Takikawa S., Kondo M., Osuka S., Mori M., Kikkawa F. (2015). Anti-Müllerian hormone levels after laparoscopic cystectomy for endometriomas as a possible predictor for pregnancy in infertility treatments. Gynecol. Endocrinol..

[140] Muzii L., Achilli C., Lecce F., Bianchi A., Franceschetti S., Marchetti C., Perniola G., Panici P.B. (2015). Second surgery for recurrent endometriomas is more harmful to healthy ovarian tissue and ovarian reserve than first surgery. Fertil. Steril..

[141] Ferrero S., Scala C., Racca A., Calanni L., Remorgida V., Venturini P.L., Maggiore U.L.R. (2015). Second surgery for recurrent unilateral endometriomas and impact on ovarian reserve: A case-control study. Fertil. Steril..

[142] Park H., Kim C.-H., Kim E.-Y., Moon J.-W., Kim S.-H., Chae H.-D., Kang B.-M. (2015). Effect of second-line surgery on in vitro fertilization outcome in infertile women with ovarian endometrioma recurrence after primary conservative surgery for moderate to severe endometriosis. Obstet. Gynecol. Sci..

[143] Kim M.-L., Kim J.M., Seong S.J., Lee S.Y., Han M., Cho Y.J. (2014). Recurrence of ovarian endometrioma after second-line, conservative, laparoscopic cyst enucleation. Am. J. Obstet. Gynecol..

[144] Beretta P., Franchi M., Ghezzi F., Busacca M., Zupi E., Bolis P. (1998). Randomized clinical trial of two laparoscopic treatments of endometriomas: Cystectomy versus drainage and coagulation. Fertil. Steril..

[145] Alborzi S., Momtahan M., Parsanezhad M.E., Dehbashi S., Zolghadri J., Alborzi S. (2004). A prospective, randomized study comparing laparoscopic ovarian cystectomy versus fenestration and coagulation in patients with endometriomas. Fertil. Steril..

[146] Seracchioli R., Mabrouk M., Frascà C., Manuzzi L., Montanari G., Keramyda A., Venturoli S. (2010). Long-term cyclic and continuous oral contraceptive therapy and endometrioma recurrence: A randomized controlled trial. Fertil. Steril..

